# Are child-centric aspects in newborn and child health systematic review and meta-analysis protocols and reports adequately reported?—two systematic reviews

**DOI:** 10.1186/s13643-017-0423-9

**Published:** 2017-03-06

**Authors:** Mufiza Farid-Kapadia, Kariym C. Joachim, Chrinna Balasingham, April Clyburne-Sherin, Martin Offringa

**Affiliations:** 1 Toronto Outcomes Research in Child Health (TORCH), Child Health Evaluative Sciences, Research Institute, The Hospital for Sick Children, University of Toronto, 686 Bay Street, Toronto, Ontario M5G 0A4 Canada; 2 Department of Paediatrics, The Hospital for Sick Children, University of Toronto, Toronto, Canada

**Keywords:** PRISMA, PRISMA-P, Systematic review, Protocol, Reporting guidelines, Child health

## Abstract

**Background:**

Evidence suggests that newborn and child health systematic reviews and meta-analyses exhibit poor quality in reporting. The “Preferred Reporting Items in Systematic Review and Meta-Analysis” (PRISMA) and PRISMA-Protocols (PRISMA-P) checklists have been developed to improve the reporting of systematic review results and protocols, respectively. We aimed to evaluate the clarity and transparency in reporting of child-centric items in child health systematic reviews (SRs) and SR protocols and to identify areas where reporting could be strengthened.

**Methods:**

Two preliminary lists of potential child-centric reporting items were used to examine current reporting. The Cochrane, DARE, MEDLINE, and EMBASE libraries were searched from 2010 to 2014 for systematic reviews that included children. Each report and protocol that met the inclusion criteria had their quality of reporting assessed by their reporting of child-centric items. Quality of reporting was assessed per whether one third, one to two thirds, or more than two thirds of papers complied with potential child-centric potential modifications/extensions to PRISMA and were analyzed by the following: (i) paper type (i.e., report vs. protocol), (ii) publication type (i.e., Cochrane vs. non-Cochrane), and (iii) population type (i.e., child-only vs. mixed populations vs. family/maternal).

**Results:**

Of the 414 eligible articles, 248 reports and 76 protocols were included. In 21 of 24 potential SR reporting items and 13 of 14 potential SR protocol reporting items, less than two thirds of papers met the child-centric reporting item requirements. Mixed population studies displayed significantly poorer reporting in comparison to child-only and family/maternal intervention studies for 11 potential SR reporting items (*p* < 0.05) and five potential SR protocol items (*p* < 0.05). When comparing non-Cochrane to Cochrane reports and protocols, five items in both lists were found to perform significantly poorer in non-Cochrane reports (*p* < 0.05). Significant differences in reporting quality were found in three of 14 items shared between the potential SR reporting items and potential SR protocol reporting items (*p* < 0.05).

**Conclusions:**

Newborn and child health systematic reviews and meta-analyses exhibit incomplete reporting, thereby hindering prudent decision-making by healthcare providers and policy makers. These results provide a rationale for the implementation of child-centric extensions and modifications to current PRISMA and PRISMA-P, such as to improve reporting in this population.

**Electronic supplementary material:**

The online version of this article (doi:10.1186/s13643-017-0423-9) contains supplementary material, which is available to authorized users.

## Background

Systematic reviews (SRs) and meta-analyses (MAs) are regarded as the highest level of medical evidence [1]. The application of rigorous methodological approaches for SRs and MAs establish the effectiveness and safety of interventions in a comprehensive, transparent, and reproducible nature [2–4]. Unfortunately, evidence shows that not all SRs are guaranteed to have been performed with the necessary methodological rigor [5–8]. Evaluations of the methodological quality of large numbers of SRs have highlighted weaknesses in their design, conduct, and reporting, even in high-impact factor journals [9–12]. Altogether, these weaknesses impair the ability to transform the results of SRs into practice and policy guidelines [9, 10].

Steps have been taken to enhance the transparency and completeness in the reporting of methods and results of the SRs. In 1996, an international group developed an SR reporting guideline called the QUOROM (QUality Of Reporting Of Meta-analyses) Statement [13], which was followed in 2009 by a guideline update and renamed to the PRISMA (Preferred Reporting Items for Systematic reviews and Meta-Analyses) Statement [14]. Furthering the transparency, consistent conduct, and replicability of SRs, the PRISMA-Protocols (PRISMA-P) Statement was created in 2015 [15]. The International Prospective Register of Ongoing Systematic Reviews (PROSPERO) together with PRISMA-P encourage the publication of SR protocols to improve processes of evidence synthesis [15]. Together, PRISMA and PRISMA-P strive to enhance the ability of stakeholders to critically appraise and replicate a SRs’ methodology, as well as to interpret its findings for use in health policy decision-making. A marked increase in the completeness in reporting of SRs has been reported in journals that endorse PRISMA [16, 17].

Despite these advances, poor reporting among current SRs in child health has repeatedly been documented [7, 18–20]. Given the large degree of physiological, pharmacological, and psychological differences among different age groups (e.g., neonates, children, adolescents, adults, elderly people) [6, 21–23], it is important that research questions be age-specific and that evidence be reported in such a way that the resulting recommendations are appropriate for each relevant age group [19]. This would need to be done for specified childhood subpopulations [20, 24], describing age-specific interventions [22, 25], comparators [26], and outcomes measures [3, 27, 28]. The failure of SRs and MAs to separately report on research aspects which are not uniformly similar across all age spectrums may impact the ability of decision-makers to inform policy and program decisions for specific age groups.

Given that the existing reporting guidelines (i.e., PRISMA and PRISMA-P) do not encompass the additional reporting complexities associated with SRs and MAs in child health, it may be prudent to expand them. While general methodological items would not be any different for child health SRs compared to those for other SRs, provision of details that pertain to child-centric features in clinical research would make the research to be useful to end-users and decision-makers. Our recent study on reporting items for child health clinical trial protocols and reports identified a number of gaps in relation to transparency, reproducibility, interpretability, internal and external validity, reporting and publication bias, accountability, scientific soundness, and research ethics [29]. Many of these issues may also hold true to child-centric SR protocols and reports. In a recent scoping review, the current authoring team of senior SR authors identified deficiencies in the reporting of child health SRs and suggested a number of potential areas where reporting could be improved [30].

Developing a child health SR reporting standard first requires assessment of the clarity and transparency in reporting the quality of published protocols and SRs to justify and inform such extensions [31, 32]. In this paper, we report two SRs that were conducted to examine the need for the development of child-centric reporting guideline extensions.

### Objective

This study aimed to evaluate the clarity and transparency in the reporting of child-centric items in child health SRs and SR protocols and to identify areas where reporting could be strengthened.

## Methods

This review was performed in accordance with the PRISMA guidelines for SRs [14] (see Additional File 1 for details) and following a defined protocol, published previously [31]. This project is not registered with PROSPERO but, instead, with the Enhancing the QUAlity and Transparency Of health Research (EQUATOR) network [33, 34].

### Search strategy

In May of 2015, a literature search of SR reports and protocols was performed. For the SR on child health SR reports and protocols, the following databases were searched for reports; the Cochrane Database of Systematic Reviews (CDSR) and the Database of Abstracts of Reviews of Effects (DARE). As both databases have been filtered to focus on SR reports, only a child search filter [35] was applied. Since DARE only includes completed SRs, not protocols, the search strategy was adjusted to identify child health SR protocols in MEDLINE and EMBASE databases. Publication date restrictions were applied from 2010 to 2014 during the literature search stage (see below), along with restriction to English language publications. Our search strategy is reported in Additional File 2.

#### Inclusion criteria

We included SR reports and protocols where the targeted population were (i) children, (ii) children and adults, and (iii) families and mothers as population only if the outcomes are measured in children. We adopted the same definitions of “systematic review” and “protocol” as set by PRISMA [14] and PRISMA-P [15], respectively. Both reviews were limited to studies published between 2010 and 2014 following the release and subsequent endorsement of the PRISMA Statement in 2009, which was a benchmark statement in improving the transparency in reporting of SRs [14]. Any reviews and protocols focusing on diagnostic evaluation or methodology were excluded, as were MAs that did not evaluate the association of an exposure/intervention with an outcome.

### Screening and full-text extraction

Potentially relevant protocols and reports were de-duplicated in Endnote X7 [36]. For the initial screening, the goal was to make sure that children were included in the titles included in this stage. One reviewer screened the title and abstract at this stage. The included records were then exported to Microsoft Excel, and a random list of titles and abstract was generated in Excel and full texts were screened until a desired sample size of at least 300 was reached. No SRs were identified not containing children as (part of) the population. A data extraction training exercise was done on 10% of the sample. Four reviewers did quadruplicate extractions; no discrepancies occurred. Then, a further 30% of the sample was double extracted; this did not yield any relevant discrepancies. Data from the final 60% of the sample was extracted by one reviewer, who had participated in all previous steps. As it was anticipated that there would be a small number of published SR protocols, it was decided that all eligible protocols identified during screening would be prioritized for full-text extraction.

#### Data collection form

The data extraction form contained three sections as follows: (i) study characteristics, (ii) whether the review or protocol fulfilled the reporting criteria in the potential child-centric modifications/extensions to PRISMA and PRISMA-P, and (iii) examples of good item reporting. Many of these details can be found within the Data Collection form in Additional File 3 and the SR Decision-Making Criteria Table in Additional File 4.

First, study characteristics were extracted, specifically the following: year of publication, country of origin, primary study design, age range of study participants, diagnoses of study participants and information on participants, intervention, comparator, and outcomes. Second, papers were evaluated on whether they reported on the potential child-centric modifications/extensions to PRISMA. Some potential extension and modification items require that certain information be extracted from single sections (e.g., Discussion) while others allow for information to be extracted from multiple sections (e.g., Abstract and/or Introductory paragraphs) of the included papers. Not every PRISMA and PRISMA-P item has a modification or a corresponding extension item, and a few have more than one potential extension per item. This means that the original PRISMA items 5, 7, 9, 10–12, 15, 17, 19, 20, and 22 and PRISMA-P items 1, 2–4, 9, 11–14, 16, and 17 do not have any potential modifications or extensions; hence, they were not evaluated in our evidence synthesis.

For these assessments, the criteria and definition for each “Yes/Incomplete/No/Not Applicable” response for each item are detailed in Additional File 4 with corresponding results. For a *modified* item to be scored as a “Yes,” both the original item of PRISMA/PRISMA-P and the potential child-centric items needed to be addressed. If either the original item or modified text was not addressed, it was considered as incomplete. The “Incomplete” responses of four modified items received further analysis to assess whether the child-centric criteria were met even if the PRISMA/PRISMA-P were not. For child-centric *extension* items, the full content of the child-centric potential modification/extension needed to be addressed in order to be evaluated as a “Yes.” Partially reported information was classified as “Incomplete.” If the item (modification or extension) was not addressed at all, it was scored as a “No.” Instances where modification and extension items did not apply to a protocol or report were thus marked as “Not Applicable.”

### Analysis

The proportion of “Yes” and “Incomplete” responses out of all eligible articles was presented for each individual item. The proportion of “No” responses would add to a total of 100% hence was not presented. Instances where items were marked as “Not Applicable” were neither counted in the numerator nor the denominator. We also provided 95% confidence intervals for each percentage based on the Wilson-Newcombe method without a continuity correction [37, 38]. Per item, quality of reporting was assessed on the basis of whether one third, one to two thirds, or more than two thirds of reviewed papers met the reporting requirements of that item. The proportion of “Yes” responses for each reporting item was further examined via two subgroup analyses, by type of review (i.e., Cochrane and non-Cochrane) and by the targeted population (child-only, mixed child and adult, and maternal/family). Pearson’s chi-squared test was used to determine whether any statistically significant differences (*p* < 0.05) existed across groups. Reports and protocols for similar items were also compared.

## Results

**Fig. 1:**
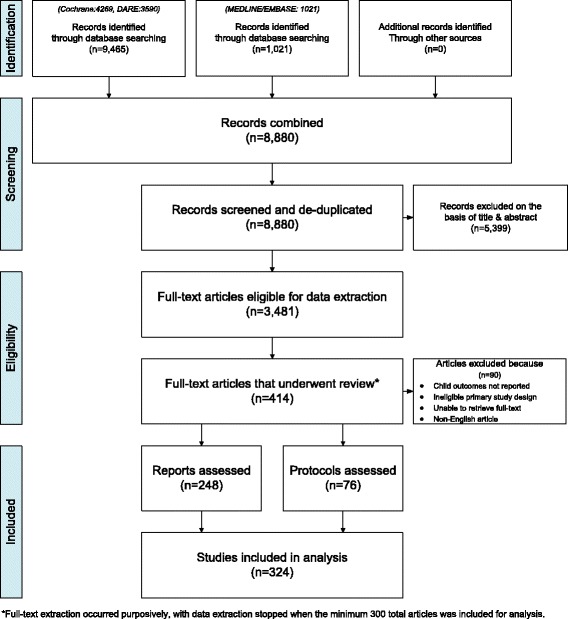
PRISMA flow diagram depicting flow of information through the different phases of both reviews

The PRISMA flow diagram in Fig. 1 demonstrates the process used to identify, screen, and select eligible reports and protocols for the SR. Our search identified a total of 8880 references (4269 from CDSR, 3590 from DARE, 1021 from MEDLINE/EMBASE). The screening of titles and abstracts identified 5399 ineligible or duplicate abstracts. All of these were excluded, leaving a set of 3481 eligible studies; the full-text articles of which were retrieved for this full set. Of the eligible set, 414 reports and protocols underwent full-text review, 90 of which did not meet the eligibility criteria. The final tally of included reports and protocols for the analysis was 324, with 248 reports and 76 protocols (see Additional File 5 for references) in total; the characteristics of which are summarized in Table 1. We present the results of each SR separately in this section.

**Table 1:** Characteristics of included newborn and child health systematic review (SR) reports and protocols

Characteristic	SR reports % (*n*)	SR protocols % (*n*)
Total number of studies	248	76
Population included		
Child-only studies	60.9 (151)	53.9 (41)
Mixed child/adult studies	25.8 (64)	31.6 (24)
Community/family/maternal intervention/exposure studies	13.3 (33)	14.5 (11)
Type		
Cochrane	59.7 (148)	34.2 (26)
Non-Cochrane	40.3 (100)	65.8 (50)
ICD-10 diagnostic category		
Infectious disease and parasitic diseases	10.5 (26)	15.8 (12)
Neoplasms (including oncology)	2.0 (5)	3.9 (3)
Blood, blood-forming organs, and the immune mechanism	1.2 (3)	2.6 (2)
Endocrine and nutritional and metabolic disease	9.7 (24)	7.9 (6)
Mental and behavioral disorders	11.3 (28)	11.8 (9)
Nervous system	4.8 (12)	6.6 (5)
Eye and adnexa	0.4 (1)	0.0 (0)
Ear and mastoid process	1.6 (4)	1.3 (1)
Circulatory system	1.2 (3)	1.3 (1)
Respiratory system	12.1 (30)	1.3 (1)
Digestive system	6.0 (15)	3.9 (3)
Skin and subcutaneous tissue	0.8 (2)	0.0 (0)
Musculoskeletal system and connective tissue	1.2 (3)	2.6 (2)
Genitourinary, childbirth, and the puerperium	11.3 (28)	3.9 (3)
Conditions originating in the perinatal period	7.7 (19)	5.3 (4)
Congenital malformation, deformations, and chromosomal abnormalities	2.4 (6)	2.6 (2)
Symptoms, signs, and abnormal clinical laboratory findings not elsewhere classified	1.2 (3)	2.6 (2)
Injury, poisoning, and consequences of external causes	5.2 (13)	7.9 (6)
External causes of morbidity and mortality	0.0 (0)	0.0 (0)
Factors influencing health status and contact with health services	0.8 (2)	10.5 (8)
Oral health	0.0 (0)	0.0 (0)
No explicit medical condition	8.1 (20)	5.3 (4)
Other	0.4 (1)	2.6 (2)
Intervention type		
Drug/natural health products	49.2 (122)	36.8 (28)
Device	8.5 (21)	2.6 (2)
Surgery or radiotherapy	7.7 (19)	7.9 (6)
Rehabilitation or psycho-social	14.5 (36)	9.2 (7)
Vaccine	0.8 (2)	2.6 (2)
Communication, organizational, or education	9.7 (24)	22.4 (17)
Prevention or screening	2.4 (6)	3.9 (3)
Complex intervention (>1 interventions)	2.4 (6)	2.6 (2)
Other	5.6 (14)	17.1 (13)
No explicit exposure or intervention	5.2 (13)	5.3 (4)
Comparison type		
Drug/natural health products	43.5 (108)	31.6 (24)
Device	7.3 (18)	2.6 (2)
Surgery or radiotherapy	4.8 (12)	5.3 (4)
Rehabilitation or psycho-social	9.7 (24)	6.6 (5)
Vaccine	0.8 (2)	2.6 (2)
Communication, organizational, or education	5.2 (13)	14.5 (11)
Prevention or screening	0.8 (2)	2.6 (2)
Complex intervention (>1 interventions)	2.0 (5)	0.0 (0)
Other	8.1 (20)	15.8 (12)
No explicit exposure or intervention	26.2 (65)	28.9 (22)

### Systematic review and meta-analysis reports

The results for our review pertaining to SR and MA *reports* are displayed in Table 2. The majority of these 248 articles (60.9%) were “child-only studies,” followed by “mixed child/adult studies” (25.8%) and “maternal/family studies” (13.3%). In addition, most of these papers were Cochrane reviews (59.7%). Pharmaceutical drugs/natural health products (including vitamin supplementation and herbal remedies) as a group were the intervention (49.2%) and/or comparator (43.5%) of interest in up to half of the SRs and MAs. SR reports generally focused on conditions affecting the respiratory system (12.1%), mental and behavioral disorders (11.3%), pregnancy/childbirth outcomes (11.3%), and infectious and parasitic diseases (10.5%).

**Table 2:** Results for newborn and child health systematic review reports

	Proportion (%) of “Yes” responses for the total (no.) of reviews eligible for scoring								
		Type of review				Intervention/exposure group			
Item^a^ number	Item	Overall (*N* = 248)	Cochrane (*N* = 100)	Non-Cochrane (*N* = 148)	*p* value	Children only (*N* = 151)	Children and adults (*N* = 64)	Family and maternal (*N* = 33)	*p* value
	Title								
1)	Identify the report as a systematic review, meta-analysis, or both **for pediatric population as a focus of review**, if applicable	62.1 (248)	67 (100)	58.8 (148)	0.194	78.8 (151)	26.6 (64)	54.5 (33)	*p* < 0.01*
	Abstract								
2)	Provide a structured summary including, as applicable, background; objectives; data sources; study eligibility criteria, including specifying **targeted pediatric age group(s)**, interventions; **primary and secondary outcomes**; study appraisal and synthesis methods; results; limitations; conclusions and implications of key findings; systematic review registration number	9.3 (248)	16 (100)	4.7 (148)	*p* < 0.01*	12.6 (151)	3.1 (64)	6.1 (33)	0.079
**2a)**	**If a systematic review includes both adults and children, describe a subgroup analysis for the targeted pediatric age group(s) in the methods and results of the abstract**	11.6 (69)	9.1 (33)	13.9 (36)	0.538	NA	11.3 (62)	14.3 (7)	0.815
**2b)**	**Describe applicability or limits of applicability of the results of the systematic review to the targeted pediatric age group(s)**	62.1 (248)	65 (100)	60.1 (148)	0.440	75.5 (151)	35.9 (64)	51.5 (33)	*p* < 0.01*
	Introduction								
	Rationale								
3)	Describe the rationale for the review in the context of what is already known								
**3a)**	**In the contexts of the synthesized evidence in adults or other pediatric groups (non-targeted), explain the rationale for synthesizing evidence for the targeted pediatric age group(s). Provide hypotheses that relate to the targeted pediatric age group(s)**	3.6 (248)	5 (100)	2.7 (148)	0.345	6 (151)	0 (64)	0 (33)	0.066
	Objectives								
4)	Provide an explicit statement of questions being addressed with reference to **targeted pediatric age groups**, interventions, comparisons, outcomes, and study design (PICOS)	0.8 (248)	2 (100)	0 (148)	0.087	0.7 (151)	1.6 (64)	0 (33)	0.703
	Methods								
	Eligibility criteria								
6)	Specify study characteristics (e.g., PICOS, length of follow-up) and report characteristics (e.g., years considered, language, publication status) used as criteria for eligibility, giving rationale								
**6a)**	**Justify the targeted pediatric age group(s) selected**	74.2 (248)	73 (100)	75 (148)	0.725	88.7 (151)	34.4 (64)	84.8 (33)	*p* < 0.01*
**6b)**	**Intervention: Justify the intervention for the targeted pediatric age group(s) addressing potential age-related differences in intervention effects**	67.7 (248)	65 (100)	69.6 (148)	0.449	78.1 (151)	32.8 (64)	87.9 (33)	*p* < 0.01*
**6c)**	**Provide rationale for extrapolation or adaptation of adult intervention, if any**	29.6 (27)	28.6 (7)	30 (20)	0.946	40 (20)	0 (7)	0 (0)	0.037
**6d)**	**Comparators: Explain the choice of comparator(s) and, if applicable, evidence for the active comparator and/or standard of care for targeted pediatric age group(s)**	9.7 (248)	11 (100)	8.8 (148)	0.564	9.9 (151)	7.8 (64)	12.1 (33)	0.759
**6e)**	**Outcomes: List and define all the primary outcomes addressed for the targeted pediatric age group(s). List and define growth and development outcomes and adverse outcomes (events), if applicable**	41.1 (248)	57 (100)	30.4 (148)	*p* < 0.01*	51.7 (151)	10.9 (64)	51.5 (33)	*p* < 0.01*
**6f)**	**Outcomes: Explain the clinical relevance of the selected outcomes (benefits and harms) for the targeted pediatric age group(s)**	34.3 (248)	38 (100)	31.8 (148)	0.312	40.4 (151)	20.3 (64)	33.3 (33)	0.018*
**6g)**	**Outcomes: Explain the validity, feasibility, and responsiveness of the outcome measures for the pre-targeted pediatric age group(s)**	0 (248)	0 (100)	0 (148)	*p* > .999	0 (151)	0 (64)	0 (33)	*p* > .999
	Search								
8)	Present full electronic search strategy for at least one database, including any limits used, such that it could be repeated								
**8a)**	**Describe the search strategy and terms (including database specific MeSH terms for pediatric population) used to address the targeted pediatric age group(s)**	44 (248)	38 (100)	48 (148)	0.124	60.3 (151)	3.1 (64)	48.5 (33)	*p* < 0.01*
	Summary measures								
13)	State the principal summary measures (e.g., risk ratio, difference in means)								
**13a)**	**If data were available for adult and pediatrics, provide summary measures for adult and targeted pediatric age group(s) separately**	2.2 (89)	2.1 (47)	2.3 (42)	0.950	NA (NA)	3.2 (63)	0 (26)	*p* < 0.01*
	Synthesis of results								
14)	Describe the methods of handling data and combining results of studies if done, including measures of consistency (e.g., I2) for each meta-analysis								
**14a)**	**For studies that included pediatrics and adults without a subgroup analysis of the pediatric population, describe how the data on targeted pediatric age group(s) were used in the analysis**	0 (89)	0 (47)	0 (42)	*p* > .999	NA (NA)	0 (63)	0 (26)	*p* > .999
	Additional analyses								
16)	Describe methods of additional analyses (e.g., sensitivity or subgroup analyses **for targeted pediatric age group(s)**, meta-regression), if done, indicating which were pre-specified	54.9 (142)	64.1 (92)	38 (50)	*p* < 0.01*	54.1 (74)	68.9 (45)	30.4 (23)	*p* < 0.01*
	Results								
	Study characteristics								
18)	For each study, present characteristics for which data were extracted (e.g., study size, PICOS, follow-up period) and provide the citations								
**18a)**	**Provide sample size of pediatric group and subgroups (if applicable) for each study.**	72.6 (234)	74.2 (89)	71.7 (145)	0.686	83.6 (146)	42.6 (61)	81.5 (27)	*p* < 0.01*
	Synthesis of results								
21)	Present results of each meta-analysis done, including confidence intervals and measures of consistency.								
**21a)**	**Report the numbers of included studies with pediatric participants. Where applicable, report the number of events and total pediatric population on which the result synthesis is based**	60.7 (168)	70.5 (78)	52.2 (90)	0.018*	79 (100)	20.4 (49)	68.4 (19)	*p* < 0.01*
	Additional analysis								
23)	Give results of additional analyses, if done (e.g., sensitivity or subgroup analyses **for the targeted pediatric age group(s)**, meta-regression [see Item 16])	54.0 (87)	65.1 (43)	43.2 (44)	0.046*	53.2 (47)	58.1 (31)	44.4 (9)	0.743
	Discussion								
	Summary of evidence								
24)	Summarize the main findings including the strength of evidence for each main outcome; consider their relevance to key groups (e.g., healthcare providers, users, **i.e., children, carer such as parents or legal guardians**, and policy makers).	74.5 (239)	70.7 (92)	76.9 (147)	0.286	83.8 (148)	48.4 (62)	82.8 (29)	*p* < 0.01*
	Limitations								
25)	Discuss limitations at study and outcome level (e.g., risk of bias, **growth and developmental outcomes in children, and minimally important differences in children) at targeted pediatric age group(s) level (e.g., newborn and infant under 5 years)**, and at review level (e.g., incomplete retrieval of identified research, reporting bias and paucity of research in children)	35.8 (232)	29.4 (85)	39.5 (147)	0.128	46.9 (145)	16.4 (61)	19.2 (26)	*p* < 0.01*
	Conclusions								
26)	Provide a general interpretation of the results in the context of other evidence (e.g., evidence from adult studies and pre-clinical studies). Implications for future research in practice, or policy **related to the targeted pediatric age group(s)**	43.9 (244)	45.8 (96)	42.6 (148)	0.624	52.7 (150)	17.5 (63)	54.8 (31)	*p* < 0.01*
	Funding								
27)	Describe sources of funding for the systematic review and other support (e.g., supply of data) and role of funders for the systematic review								
**27a)**	**For each included trial in a systematic review, indicate (a plan to include) the source of financial support (such as government, academia or industry), if any, in the trial(s)**	5.6 (248)	8 (100)	4.1 (148)	0.190	4 (151)	10.9 (64)	3 (33)	0.113

*p-value correspondent to statistical significant at < 0.05

^a^Item numbers are potential neonatal and child health modification (bold) and extension (a–g) items for PRISMA-P

#### Title and abstract

Under two thirds (62.1%; 95% confidence interval 56 to 68%) of SRs/MAs, explicitly identified themselves as such in the *title*, while also stating their targeted pediatric age group (Item 1). Child-only studies were significantly more likely to declare this information than family/maternal studies and mixed child/adult studies (78.8%; 71.6 to 84.6% vs. 54.5; 37.9 to 70.1 and 26.6%; 17.3 to 38.5% *p* < 0.01).

A minority of papers (9.3%; 6.3 to 13.6%) (i) fulfilled the original PRISMA recommendations (provide background, objectives, data sources, etc.), (ii) specified the targeted pediatric age group (using a unit of time such as weeks, months, or years), and (iii) stated their primary and secondary outcome(s) (Item 2). Cochrane papers, however, were more likely to comply with this recommendation than non-Cochrane papers (16.0%; 10.1 to 24.4 vs. 4.7%; 2.3 to 9.4% *p* < 0.01).

In mixed child/adult reports, less than a third of articles (11.6%; 6.0 to 21.3%) provided a structured description of a subgroup analysis for their targeted pediatric age group (Item 2a). With respect to statements on the applicability of their results to their targeted pediatric age group(s), 62.1%; 55.9 to 67.9% of studies met this item (Item 2b). Of the three intervention groups, however, we found that child-only studies were significantly more likely to state the limits of applicability than mixed child/adult studies and family/maternal studies (75.5%; 68.1 to 81.7 vs. 35.9%; 25.3 to 48.1 vs. 51.5%; 35.2 to 67.5%, *p* < 0.01)

#### Introduction

Only 3.6%; 1.9 to 6.7% of reports provided a rationale for synthesizing evidence for the targeted pediatric age group(s) and providing hypotheses related to said group(s) (Item 3a). When reports were not required to state hypotheses, reporting improved—with more than two thirds of papers and then meeting this item’s requirements (64.5%; 58.4 to 70.2%). Very few papers (0.8%; 0.2 to 2.9%) stated their targeted pediatric age group (in units of time) within an explicit objective(s) statement (Item 4).

#### Methods

Nearly all items reported less than one- or two thirds compliance, except for two potential child-centric items which received more than two thirds compliance in the literature.

##### Eligibility criteria

Within the *Methods*, PRISMA encourages SRs and MAs to unambiguously describe the eligibility criteria used in the review—stating important details such as study characteristics (e.g., PICOS, length of follow-up) and report characteristics (e.g., years considered, language, publication status), while providing a rationale. More than two thirds of papers (74.2%; 68.4 to 79.2%) justified choosing their selected pediatric age group (Item 6a). Overall, however, child-only and family/maternal studies were more apt to meet this expectation than mixed child/adult studies (88.7%; 82.7 to 92.8%, 84.8%; 69.0 to 93.3 vs. 34.4%; 23.9 to 46.6% *p* < 0.01).

More than two thirds of reports (67.7%; 61.6 to 73.2%) justified the intervention for the targeted age group while addressing age-related differences in intervention effects (Item 6b), while less than one third provided a rationale for extrapolating or adapting adult interventions if done (29.6%; 15.8 to 48.5%, Item 6c). Child-only studies and family/maternal studies performed considerably better at justifying interventions than mixed child/adult studies (87.9%; 72.7 to 95.2 and 78.1%; 70.9 to 84.0 vs. 32.8%; 22.6 to 45.0% *p* < 0.01). While 40.0%; 21.9 to 61.3% of child-only reports discussed rationales for adapting adult interventions for their targeted pediatric age group; no mixed child/adult studies or family/maternal studies fulfilled this criterion (*p* = 0.037).

Less than a tenth (9.7%; 6.6 to 14.0%) of papers provided justification of the selected comparators for targeted pediatric age groups (Item 6d). Primary outcomes were listed and defined in less than half of reports (41.1%; 35.2 to 47.3%, Item 6e) though Cochrane papers were significantly more likely to fulfill this goal than non-Cochrane papers (57%; 47.2 to 66.3 vs. 30.4%; 23.6 to 38.2% *p* < 0.01). Child-only studies and family/maternal studies were significantly more likely to complete this requirement than mixed child/adult studies as well (51.7%; 43.8 to 59.5 and 51.5%; 35.2 to 67.5% vs. 10.9%; 5.4 to 20.9% *p* < 0.01).

The clinical relevance of selected outcomes for the targeted pediatric age groups was discussed in a third of papers (34.3%; 28.7 to 40.4%, Item 6f), while the validity, feasibility, and responsiveness were not discussed together in any of the published papers (Item 6g). Only when stratifying by population did we find that clinical relevance was most often discussed in child-only studies, followed by family/maternal studies, and discussed the least in mixed child/adult studies (40.4%; 32.9 to 48.4% vs. 33.3; 19.7 to 50.36 vs. 20.3%; 12.3 to 31.7% *p* = 0.018).

##### Search strategy

The potential child-centric reporting items could recommend that reports describe their search strategy and corresponding terms within the *Methods* (Item 8a), whether it includes database-specific MeSH terms for pediatric populations used to address the targeted pediatric age group(s), and whether OR is used (to enhance sensitivity) or AND (to increase specificity). Less than half of studies included child-centric search terms (44.0%; 38.0 to 50.2%), with child-only studies most likely to include these search terms (60.3%; 52.3 to 67.8%) followed by family/maternal studies (48.5%; 32.5 to 64.8%) and mixed child/adult studies being least likely to do so (3.1%; 0.9 to 10.7%) (*p* < 0.01).

##### Summary measures and synthesis of results

In SRs and MAs where eligible studies include both adults and children, our potential child-centric modifications/extensions to PRISMA recommend that authors provide summary measures for the adult and targeted pediatric age group(s) separately (Item 13a). Identified reports presented far less than one-third compliance with this item, with only 2.2%; 0.6 to 7.8% of papers meeting this recommendation. Mixed child/adult studies were more likely to meet this recommendation than family/maternal studies (3.2%; 0.9 to 10.9 vs. 0%; 0 to 12.9%, *p* < 0.01).

For reviews that include studies with mixed/child populations yet did not present pediatric results separately, our potential child-centric modifications/extensions to PRISMA recommend that reports provide justification for why the data for adult and children were combined (Item 14a); none of the reports met this recommendation.

##### Additional analyses

The last recommendation of our potential child-centric modifications/extensions to PRISMA concerning *Methods* reporting is to describe whether analyses relating to the target pediatric age groups were done and state the reasons why or why not (Item 16). Less than two thirds of papers met this item, with 54.9%; 46.7 to 62.8% of papers meeting the recommendation. Cochrane reviews, however, were significantly more likely to fulfill this criterion than non-Cochrane reviews (64.1%; 53.9 to 73.2 vs. 38%; 25.9 to 51.8%, *p* < 0.01). Mixed child/adult studies most often stated their plan to do a subgroup analysis for their targeted pediatric age group (68.9%; 54.3 to 80.5%), followed by child-only studies (54.1%; 42.8 to 65.0%); with family/maternal studies being least likely to do so (30.4%; 15.6 to 50.8%) (*p* < 0.01).

#### Results

##### Study characteristics

Each study in a SR or MA for which data has been extracted should have their characteristics (e.g., study size, PICOS, follow-up period) presented alongside the citations (Item 18a). Our potential child-centric modifications/extensions to PRISMA further recommend that the sample size of each pediatric group and subgroup be stated. Reporting of this aspect of the potential child-centric PRISMA extension was found in 72.6%; 66.6 to 77.9% of reports. Child-only studies and family/maternal studies were most likely to do this compared to mixed child/adult studies (83.6%; 76.7 to 88.7%, 81.5%; 63.3 to 91.8 vs. 42.6%; 31.0 to 55.1%, *p* < 0.01).

##### Synthesis of results

For MAs, it is recommended that the confidence intervals and measures of consistency be presented. In addition, our potential child-centric modifications/extensions to PRISMA purport that the number of included studies with pediatric participants, the number of events concerning children, and the total pediatric population on which the results synthesis is based, including age-subgroups, be stated as well (Item 21a). Here, 60.7%; 53.2 to 67.8% of reports meet this criterion. Cochrane papers were significantly more likely to meet this reporting recommendation (70.5%; 59.6 to 79.5%) than non-Cochrane papers (52.2%; 42.0 to 62.2%, *p* = 0.018). Child-only studies and family/maternal studies were more likely to meet this recommendation than mixed child/adult studies (79.0%; 70.0 to 85.8% and 68.4%; 46.0 to 84.6 vs. 20.4; 11.5 to 33.6%, *p* < 0.01).

##### Additional analyses

Regarding the propensity for which papers reported the results of additional analyses for their targeted pediatric age groups (Item 23), 54.0%; 43.6 to 64.1% of papers met this recommendation. Cochrane reviews and MAs were significantly more likely to report the results of additional analyses than non-Cochrane papers (65.1%; 50.7 to 77.2% vs. 43.2%; 29.7 to 57.8% *p* = 0.046).

#### Discussion

##### Summary of evidence

In general, 74.5%; 68.6 to 79.6% of studies are summarizing the main findings of each of their main outcomes and considering their relevance to key stakeholders such as healthcare providers, users (i.e., children and their families), and policy makers (Item 24). Compared to mixed child/adult studies (48.4%; 36.4 to 60.6%), child-only studies (83.8%; 77.0 to 88.9%) and family/maternal studies (82.8%; 65.5 to 92.4%) performed significantly better (*p* < 0.01).

##### Limitations

Overall, 35.8%; 29.9 to 42.2% of studies discussed their limitations at the included study, child health outcome and review levels (e.g., lack of research in children, lack of growth, and development outcomes) (Item 25), with child-only studies (46.9%; 39.0 to 55%) performing significantly better than family/maternal studies (19.2%; 8.5 to 37.8%) and mixed child/adult studies (16.4%; 9.2 to 27.6%) (*p* < 0.01).

#### Conclusions

The penultimate potential child-centric modification/extension recommendation is that concluding statements give a general interpretation of the results in the context of “other evidence” (e.g., evidence from adult studies and pre-clinical studies) and state implications for future research in practice or policy related to the targeted pediatric age group(s) (Item 26). Overall, this was found in 43.9%; 37.8 to 50.2% of reports. Less than two thirds of family/maternal studies (54.8%; 37.7 to 70.8%), and child-only studies (52.7%; 44.7 to 60.5%), fulfilled this requirement, while less than one third of child/adult studies (17.5%; 10.1 to 28.7%) met this expectation (*p* < 0.01).

#### Funding

Finally, authors are asked to include the source of financial support for each of the studies they included in their SR/MA (Item 27a). Overall, the proportion of papers meeting this recommendation was very low at 5.6%; 3.4 to 9.2%.

### Systematic review and meta-analysis protocols

The results of our review pertaining to SR and MA *protocols* are displayed in Table 3. Data for this SR was extracted from 76 eligible protocols. A majority of these addressed child-only intervention/exposure groups (53.9%) followed by mixed child/adult groups (31.6%); the majority of eligible protocols (65.8%) were non-Cochrane in origin, while over one third of protocols focused on pharmaceutical drugs and natural health products as the intervention/comparator of interest.

**Table 3:** Results for newborn and child health systematic review protocols

	Proportion (%) of “Yes” responses for the total (no.) of reviews eligible for scoring								
			Type of review			Intervention/exposure group			
Item^a^ number	Item	Overall (*N* = 76)	Cochrane (*N* = 50)	Non-Cochrane (*N* = 26)	*p* value	Children only (*N* = 41)	Children and adults (*N* = 24)	Family and maternal (*N* = 11)	*p* value
	**Title**								
1)	Identify the protocol as a systematic review, meta-analysis, or both **for pediatric population as a focus of review**, if applicable	72.4 (76)	88.5 (50)	64 (26)	0.015*	92.7 (41)	29.2 (24)	90.9 (11)	*p* < 0.01*
	Support								
5a)	Indicate sources of financial or other support for the review								
5b)	Provide name for the review funder and/or sponsor								
5c)	Describe roles of funder(s), sponsor(s), and/or institution(s), if any, in developing the protocol								
**5d)**	**For each included trial in a systematic review, indicate (a plan to include) the source of financial support (such as government, academia, or industry), if any, in the trial(s)**	13.2 (76)	30.8 (50)	4 (26)	0.010*	19.5 (41)	8.3 (24)	0 (11)	0.178
	**Introduction**								
	Rationale								
6)	Describe the rationale for the review in the context of what is already known								
**6a)**	**In the contexts of the synthesized evidence in adults or other pediatric groups (non-targeted), explain the rationale for synthesizing evidence for the targeted pediatric age group(s). Provide hypotheses that relate to the targeted pediatric age group(s)**	1.3 (76)	0 (50)	2 (26)	0.321	2.4 (41)	0 (24)	0 (11)	0.682
	Objectives								
7)	Provide an explicit statement of questions the review will address with reference to **targeted pediatric age groups**, interventions, comparisons, outcomes, and study design (PICOS).	0 (76)	0 (50)	0 (26)	*p* > 0.999	0 (41)	0 (24)	0 (11)	*p* > 0.999
	Methods								
	Eligibility criteria								
8)	Specify the study characteristics (such as PICO, study design, setting, time frame) and report characteristics (such as years considered, language, publication status) to be used as criteria for eligibility for the review								
**8a)**	**Justify the targeted pediatric age group(s) selected.**	64.5 (76)	84.6 (50)	54 (26)	*p* < 0.01*	78 (41)	45.8 (24)	54.5 (11)	0.022*
**8b)**	**Intervention: Justify the intervention for the targeted pediatric age group(s) addressing potential age-related differences in intervention effects**	55.3 (76)	76.9 (50)	44 (26)	*p* < 0.01*	61 (41)	37.5 (24)	72.7 (11)	0.075*
**8c)**	**Provide rationale for extrapolation or adaptation of adult intervention, if any**	16.7 (18)	20 (13)	15.4 (5)	0.826	33.3 (6)	9.1 (11)	0 (1)	0.410
**8d)**	**Comparators: Explain the choice of comparator(s) and, if applicable, evidence for the active comparator and/or standard of care for targeted pediatric age group(s)**	17.1 (76)	26.9 (50)	12 (26)	0.142	26.8 (41)	8.3 (24)	0 (11)	0.050
**8e)**	**Outcomes: List and define all the primary outcomes addressed for the targeted pediatric age group(s). List and define growth and development outcomes and adverse outcomes (events), if applicable**	55.3 (76)	80.8 (50)	42 (26)	*p* < 0.01*	78 (41)	12.5 (24)	63.6 (11)	*p* < 0.01*
**8f)**	**Outcomes: Explain the clinical relevance of the selected outcomes (benefits and harms) for the targeted pediatric age group(s)**	28.9 (76)	23.1 (50)	32 (26)	0.405	26.8 (41)	29.2 (24)	36.4 (11)	0.797
**8g)**	**Outcomes: Explain the validity, feasibility, and responsiveness of the outcome measures for the pre-targeted pediatric age group(s)**	0 (76)	0 (50)	0 (26)	*p* > 0.999	0 (41)	0 (24)	0 (11)	*p* > 0.999
	Search strategy								
10)	Present draft of search strategy to be used for at least one electronic database, including planned limits, such that it could be repeated								
**10a)**	Describe the search strategy and terms (including database specific MeSH terms for pediatric population) used to address the targeted **pediatric age group(s)**	48.7 (76)	53.8 (50)	46 (26)	0.520	58.5 (41)	25 (24)	63.6 (11)	0.017*
	Data synthesis								
15b)	If data are appropriate for quantitative synthesis, describe planned summary measures, methods of handling data and methods of combining data from studies, including any planned exploration of consistency (such as I2, Kendall’sτ)								
**15b)**	**For studies that included pediatrics and adults without a subgroup analysis of the pediatric population, describe how the data on targeted pediatric age group(s) were used in the analysis**	0 (27)	0 (21)	0 (6)	*p* > 0.999	0 (0)	0 (24)	0 (3)	*p* > 0.999
	Additional analyses								
15c)	Describe methods of additional analyses (e.g., sensitivity or subgroup analyses **for targeted pediatric age group(s)**, meta-regression), if done, indicating which were pre-specified	61 (59)	58.3 (35)	62.9 (24)	0.730	53.3 (30)	68.4 (19)	70 (10)	0.454

*p-value correspondent to statistical significant at < 0.05

^a^Item numbers are potential neonatal and child health modification (bold) and extension (a–g) items for PRISMA-P

Methodological quality was planned for assessment in nearly all protocols (96.1%), though publication bias was only planned to be assessed in about half of the eligible protocols (52.6%). A suitable number of conditions and diseases were represented in this sample, with the largest group of protocols focusing on infectious and parasitic diseases (15.8%), mental and behavioral disorders (11.8%), and factors influencing health status and contact with health services (10.5%).

#### Title

Title requirements (i.e., Item 1: identify the protocol as a SR, MA, or both for the pediatric population as a focus of review were met in 72.4%; 61.5 to 81.2% of protocols. Cochrane protocols were most likely to have complete titles (88.5%; 76.8 to 94.7%) than non-Cochrane protocols (64.0%; 44.8 to 79.5%) (*p* = 0.015). Child-only protocols and family/maternal protocols were significantly more likely to meet this recommendation than mixed child/adult protocols (92.7%; 80.6 to 97.5%, 90.9%; 62.3 to 98.4 vs. 29.2%; 14.9 to 49.2%, *p* < 0.01).

#### Support

The proportion of protocols which planned to provide information on the source of financial support for each included trial (Item 5d) was small—with 13.2%; 7.3 to 22.6% of protocols intending to provide this information. This requirement was significantly more likely to be met by protocols within the Cochrane database than that within non-Cochrane protocols (30.8%; 19.8 to 44.6 vs. 4.0%; 0.7 to 19.1%, *p* = 0.01).

#### Introduction

##### Rationale and objectives

Protocols, much like reviews, did not tend to provide a rationale for synthesizing evidence in their targeted pediatric age group nor provide an accompanying hypothesis (Item 6a). This item was met in less than one third of protocols, with 1.3%; 0.2 to 7.1% meeting this recommendation. When the requirement to state hypotheses was rescinded, 87.6%; 78.3 to 93.2% conformed to this item, considerably improving reporting. All protocols failed to state their targeted pediatric age group (in units of time) within an explicit objective(s) statement (Item 7).

#### Methods

Overall, *Methods* sections of protocols saw one to two thirds compliance, except for one child-centric potential modification for PRISMA-P. Protocols failed to adequately state vital details of and provide a rationale for each of the choices made in the design of the study.

##### Eligibility criteria

In 64.5%; 53.3 to 74.3% of protocols, the targeted pediatric age group was justified (Item 8a). Cochrane database protocols (84.6%; 72.2 to 92.1%) were more likely to meet this item than non-Cochrane protocols (54.0%; 35.6 to 71.4%) (*p* < 0.01). Child-only protocols (78.0%; 63.2 to 88.0%) were most likely to meet this recommendation, followed by family/maternal studies (54.5%; 28.0 to 78.7%), while mixed child/adult studies (45.8%; 27.9 to 64.9%) met this criterion least often (*p* = 0.022). Justification for the intervention for the targeted pediatric age group (Item 8b) was found in 55.3%; 44.1 to 66.0% of protocols. Cochrane database protocols (76.9%; 63.6 to 86.4%) were more likely to meet this criterion than non-Cochrane protocols (44.0%; 26.9 to 62.6%) (*p* < 0.01). Overall, we found that when done, only 16.7%; 5.9 to 39.3% of eligible protocols provided a rationale for extrapolating or adapting adult interventions for children (Item 8c), and that, only 17.1%; 10.3 to 27.1% of protocols explained their choice of comparator and provided evidence for the usage of that comparator in a targeted pediatric age group (Item 8d).

Less than two thirds of protocols reported primary outcomes and their definition (Item 8e), with just over half of protocols (55.3%; 44.1 to 66.0%) presenting their outcomes in this manner. Cochrane protocols were significantly more likely to fulfill this need (80.8%; 67.9 to 89.4%) than non-Cochrane protocols (42.0%; 25.3 to 60.8%) (*p* < 0.01). Child-only protocols and family/maternal protocols outperformed mixed child/adult protocols (78%; 60.05 to 85.7 and 63.6%, 35.1 to 84.6 vs. 12.5%; 4.3 to 31.0% *p* < 0.01). The clinical relevance of selected outcomes for the targeted pediatric age groups was discussed (Item 8f) in under a third of papers (28.9%; 19.9 to 39.9%), while the validity, feasibility, and responsiveness were not discussed together (Item 8g) in any of the published papers.

##### Search strategy

With respect to the issue that protocols detail a draft of their search strategy for at least one electronic database, detailing whether it includes database specific MeSH terms for pediatric populations used to address the targeted pediatric age group(s), and whether OR is used (to enhance sensitivity), or AND (to increase specificity), (our Item10a), less than half (48.7%; 37.8 to 59.7%) of protocols provided a search strategy with child-centric search terms. When comparing intervention groups, however, child-only intervention protocols (63.6%; 35.3 to 84.8%) and family/maternal protocols (58.5%; 43.3 to 72.2%) were significantly more likely to meet this recommendation than mixed child/adult protocols (25.0%; 12.0 to 44.9%) (*p* = 0.017).

##### Data synthesis and additional analyses

Wherever a proposed SR or MA could potentially include studies featuring child and adult populations without subgrouping, our potential child-centric modifications/extensions to PRISMA-P also recommend that the protocol states how that data would be analyzed (Item 15b). As with evidence synthesis, we found that none of the protocols met this recommendation. Finally, where any additional analyses were to be performed regarding a pediatric age group, our potential child-centric modifications/extensions to PRISMA-P call for a description of how the sub-analyses are to be performed (Item 15c). This item was considered fulfilled in 61%; 48.2 to 72.4% of protocols.

### Systematic reviews reports vs. systematic review protocols

Table 4 compares the reporting of SR reports and protocols when assessed by the child-centric potential modifications/extensions to PRISMA and PRISMA-P. We found there were only three areas where there was a statistically significant difference between the reporting quality of reports and protocols. First, reports were more likely to justify the intervention for the targeted pediatric age group(s) (while addressing potential age-related differences in intervention effects (potential PRISMA Item 6b/potential protocol PRISMA Item 8b) (67.7%; 61.6 to 73.2 vs. 55.3%; 44.1 to 66.0%, *p* = 0.047). Second, protocols were more likely to list and define all primary outcomes for the targeted pediatric age group (potential Item 6e/potential protocol Item 8e) (55.3%; 44.1 to 66.0 vs. 41.1%; 35.2 to 47.3% *p* = 0.031). Finally, protocols were more likely to report (or plan to report) the sources of funding for each trial within the SR or MA (potential Item 27a/potential protocol Item 5a) (13.2%; 7.4 to 22.6 vs. 5.6%; 3.4 to 9.2% *p* = 0.03).

**Table 4:** Comparison of overall reporting quality of newborn and child health systematic review (SR) reports vs. protocols

	Proportion (%) of “Yes” responses for the total (no.) of reviews eligible for scoring			
Item^a^ number	Item	SR reports (*N* = 248)	SR protocols (*N* = 76)	*p* value
	**Title**			
1/1a)	Identify the report as a systematic review, meta-analysis, or both **for pediatric population as a focus of review**, if applicable	62.1 (248)	72.4 (76)	0.102
	**Introduction**			
	Rationale			
5/6)	Describe the rationale for the review in the context of what is already known			
	**In the contexts of the synthesized evidence in adults or other pediatric groups (non-targeted), explain the rationale for synthesizing evidence for the targeted pediatric age group(s). Provide hypotheses that relate to the targeted pediatric age group(s)**	3.6 (248)	1.3 (76)	0.309
	Objectives			
4/7)	Provide an explicit statement of questions being addressed with reference to **targeted pediatric age groups**, interventions, comparisons, outcomes, and study design (PICOS)	0.8 (248)	0 (76)	0.433
	**Methods**			
	Eligibility criteria			
6/8)	Specify study characteristics (e.g., PICOS, length of follow-up) and report characteristics (e.g., years considered, language, publication status) used as criteria for eligibility, giving rationale			
**6a/8a)**	**Justify the targeted pediatric age group(s) selected**	74.2 (248)	64.5 (76)	0.1
**6b/8b)**	**Intervention: Justify the intervention for the targeted pediatric age group(s) addressing potential age-related differences in intervention effects**	67.7 (248)	55.3 (76)	0.047*
**6c/8c)**	**Provide rationale for extrapolation or adaptation of adult intervention, if any**	29.6 (27)	16.7 (18)	0.331
**6d/8d)**	**Comparators: Explain the choice of comparator(s) and, if applicable, evidence for the active comparator and/or standard of care for targeted pediatric age group(s)**	9.7 (248)	17.1 (76)	0.076
**6e/8e)**	**Outcomes: List and define all the primary outcomes addressed for the targeted pediatric age group(s). List and define growth and development outcomes, adverse outcomes (events), if applicable**	41.1 (248)	55.3 (76)	0.031*
**6f/8f)**	**Outcomes: Explain the clinical relevance of the selected outcomes (benefits and harms) for the targeted pediatric age group(s)**	34.3 (248)	28.9 (76)	0.388
**6g/8g)**	**Outcomes: Explain the validity, feasibility and responsiveness of the outcome measures for the pre-targeted pediatric age group(s)**	0 (248)	0 (76)	*p* > 0.999
	Search			
8/10)	Present full electronic search strategy for at least one database, including any limits used, such that it could be repeated			
**8a/10a)**	**Describe the search strategy and terms (including database specific MeSH terms for pediatric population) used to address the targeted pediatric age group(s)**	44 (248)	48.7 (76)	0.469
	Synthesis of results			
14/15b)	Describe the methods of handling data and combining results of studies, if done, including measures of consistency (e.g., I2) for each meta-analysis			
**14a/15b)**	**For studies that included pediatrics and adults without a subgroup analysis of the pediatric population, describe how the data on targeted pediatric age group(s) were used in the analysis**	0 (89)	0 (27)	*p* > 0.999
	Additional analyses			
16/15c)	Describe methods of additional analyses (e.g., sensitivity or subgroup analyses **for targeted pediatric age group(s)**, meta-regression), if done, indicating which were pre-specified	54.9 (142)	61 (59)	0.429
	**Funding**			
27/5)	Describe sources of funding for the systematic review and other support (e.g., supply of data) and role of funders for the systematic review			
**27a/5a)**	**For each included trial in a systematic review, indicate (a plan to include) the source of financial support (such as Government, Academia or Industry), if any, in the trial(s)**	5.6 (248)	13.2 (76)	0.03*

*p-value correspondent to statistical significant at < 0.05

^a^Item numbers are potential neonatal and child health modification (bold) and extension (a–g) items for PRISMA-C/PC

## Discussion

We found a relatively incomplete reporting of child-centric study details across SRs and MAs on intervention studies in children published between 2010 and 2014. Studies with mixed child and adult populations were significantly more likely to demonstrate incomplete reporting relative to other groups. In SRs and MAs where both children and adults are to be included, it appears necessary that children be analyzed separately in subgroups. Our results reaffirmed previous evidence that Cochrane reviews uphold a higher standard of reporting over other SR publications in general. We did not find significant differences in reporting quality between SR reports and protocols. There are currently no child-centric guidelines that can account for the complexities associated with the design, implementation, and reporting of SRs and MAs in neonates and children [31]. Taken together, we identified a large degree of incomplete reporting of both reports and protocols—thereby highlighting the need for child-centric reporting guidelines. The results of this work provide an evidence foundation for developing child-centric PRISMA-C and PRISMA-PC guidelines.

### Overall quality and completeness of reporting

Incomplete reporting on child-centric topics was a trend across all reports and protocols, with SR *titles* being the only aspect of reporting to be seen in more than two thirds of papers. Overall, *Abstract* and *Introduction* sections were consistently incomplete, while *Methods* were more heterogeneous. Regardless of whether reports or protocols were being assessed, the majority of the *Methods* sections were incompletely reported on child-centric topics. *Results* and *Discussions* were slightly better. Reporting on funding was uncommon. A recent evaluation of the quality of reporting of SRs identified that while the number of published SRs has increased threefold since 2004, the overall quality of reporting have not improved substantially [39].

Our findings are consistent with earlier studies that demonstrated incomplete reporting quality of SR abstracts in general or adult populations, whereas the directionality of a determinant-outcome effect could not be determined from one in four abstracts from the general and specialty medical literature [40]. The dearth of quality reporting in research in children has been observed numerous times, yielding repeated calls for child-centric reporting guidelines in the literature [3, 18, 19, 29]. A similar conclusion was reached in a SR assessing issues in the design, reporting, and conduct of clinical trials in children [29]. To address these challenges, 8 and 14 child-centric items were added to the Standard Protocol Items for Randomized Trials in Children (SPIRIT-C) and Consolidated Standards of Reporting Trials in Children (CONSORT-C), respectively [29]. Taken together, our findings indicate that overall reporting can be improved with the following areas needing improvement the most: (i) adequate description of the targeted pediatric (sub)populations in the *Abstract*; (ii) explicit rationales and objectives for the targeted pediatric age groups in *Introduction*; and (iii) details on how data on various targeted pediatric age groups are analyzed in *Methods* and *Results*. Finally, provision of information on the sources of financial support for each trial in the SR would be helpful. Improving reporting among these areas for improvement would also reflect an improvement in the methodological quality of SRs and MAs. This is acknowledged by the PRISMA Statement—when assessing study quality, the distinction between reporting and methodological quality is less straightforward for SRs than for individual studies because the reporting and conduct of SRs are closely intertwined [14].

### Quality of reporting by population

Of the intervention/exposure groups investigated throughout this review, mixed population studies that include both children and adults were the least likely to explicitly report their work for children and adults. In most reporting areas, mixed child/adult studies showed less than one third compliance, while child-only and family/maternal groups performed considerably better. For example, among both reports and protocols, mixed child/adult studies were the least likely to list and define all the primary outcomes addressed for their targeted pediatric age groups. Also, among the eligible reports, mixed child/adult SRs were least likely to report the sample sizes of the pediatric subgroups for each included study. These results are similar to those in a report showing it was not possible to determine the ages of trial participants in a large proportion (35%) of SRs [20]. Inadequate reporting of age groups hinders the ability to draw inferences about the different age groups based on the synthesized evidence [41]. Altogether, these results suggest a lack of awareness in the literature on the fundamental dissimilarities between children and adults and the importance of differentiating between age and developmental strata when performing research. To ensure that age-related differences are accounted for, reports and publications that include mixed populations would benefit the most from child-centric reporting guidelines.

### Quality of reporting in Cochrane Reviews

Though overall reporting quality remained low, we found somewhat better quality reporting in Cochrane reports and protocols. In general, reporting of Cochrane reports varied, with instances of better reporting in the *Abstract*, *Methods*, and *Results* sections. Cochrane protocols have strong *Titles*, *Funding information*, and *Methods* sections. However, for most of the potential child-centric modifications/extensions items, however, there were no statistically significant differences between Cochrane and non-Cochrane reviews. Reports of higher quality reporting in Cochrane reviews are not new; when using PRISMA, QUORUM, and AMSTAR to assess reporting quality, Cochrane reviews were found to perform well in general [39] as well as in numerous health disciplines and age groups, including neonatology [42], orthodontics [43], otolaryngology [44], physiotherapy [45], and pediatric dentistry [46, 47]. Taken together, while there is evidence of better reporting, there is still much room for improvement within Cochrane child health SR reports and protocols overall.

### Quality of reporting between protocols and reports

Reporting quality of SR reports and protocols was generally the same, though protocols performed better in two important aspects of the potential child-centric modifications/extensions to PRISMA; protocols were significantly more likely to justify interventions for the targeted pediatric age groups and to list and define primary outcomes addressed for the targeted pediatric age groups. While the prevalence of funding source reporting was low in reports, protocols were more likely to state their intention to report this information than reports. Further subgroup analysis of protocols found that Cochrane protocols stated their intention to report this information more often than non-Cochrane protocols. This suggests that while there is still considerable room for improvement, we may see a growing percentage of reports with this information included, especially if the Cochrane reports follow the plans laid out in their protocols.

In summary, the results of our reviews confirm a need for child health specific reporting extensions for PRISMA and PRISMA-P with modifications and new items in most areas of these two existing reporting guidelines. The results of this review provide a knowledge foundation for the development of these topics in the existing reporting standards, in the form of (i) *Modifications*, (i.e., original items from the PRISMA-P or PRISMA checklists that have been modified in wording to incorporate child-centric aspects of reporting) and (ii) *Extensions*, (i.e., new items that do not exist in the original PRISMA/PRISMA-P checklists and are included as additional child-centric components for the original items). The results can also inform accompanying Explanation and Elaboration (E&E) documents. Our next step is therefore to undertake a comprehensive consensus exercise with methodologists, systematic reviewers, clinicians, and journal editors with a special interest in child health SRs to develop these guidelines.

While the development of these extensions themselves seems worthwhile, the process has to include a serious exploration of avenues to enhance the extent to which reporting guidelines are likely to actually improve reporting. As current evidence suggests that PRISMA has had only a marginal effect on reporting quality of SRs [39], additional strategies that improve reporting are needed. These could include an update of the Cochrane Handbook, pro-active engagement of SR funders and journal editors publishing SRs, and learned societies and guideline developers to endorse the use of reporting standards, all to reduce research waste.

### Strengths and limitations

This work has several strengths. Both of our reviews include published studies from four major databases (i.e., Cochrane, DARE, MEDLINE, and EMBASE). By including a variety of papers from a range of disease areas, interventions, exposures, and patient populations, we believe that we have presented results that are reflective of the quality of current reports and protocols of SRs and MAs including children. Our findings of incomplete reporting therefore appear generalizable to all SRs and MAs that include pediatric age groups. Second, by relying on PRISMA and PRISMA-P as the basis for quality assessment and improvement, we further justified the need for and legitimacy of these two guidelines while building a rationale for improved reporting in children.

A limitation, however, could be the restriction of our search strategy to SRs and protocols published in English between the years 2010 and 2014. We are therefore unable to state the effects of language on reporting quality. We selected the 2010–2014 time span because we wanted to observe the current reporting quality after the original PRISMA Statement on reporting quality was released in 2009. Moreover, we searched only CDSR and DARE for SRs, which is likely to yield higher quality SRs than those indexed elsewhere. Yet, it highlights the need for a reporting standard, given the overall incomplete reporting. Another limitation of our reviews is the inherent subjectivity that lies in assessing the reporting quality of each report and protocol. Though the reviewers were guided by the original PRISMA and PRISMA-P guidelines as well as extension and modification items for child relevant SR protocols and reports, it was challenging to ensure that reports and protocols received consistent scoring both between reviews and over the course of data extraction. To account for these difficulties, we operationalized the requirements for a “Yes,” “No,” “Incomplete,” and “Not applicable” score for each modification and extension item (Additional File 4). We piloted and reviewed the scoring of the first 10% of the studies to ensure consistency of reviewers’ classification of each item and discussed any discrepancy in classification.

## Conclusions

Newborn and child health SRs and MAs exhibit incomplete reporting of child-centric topics. Reports and protocols with a mixed children/adult population are more prone to incomplete reporting than child-only populations, while Cochrane reviews are of a better reporting standard than non-Cochrane reviews. Overall, these results provide a rationale for increased adoption of the PRISMA and PRISMA-P reporting guidelines and a basis for child-centric extensions and modifications to these reporting standards to improve the ability of decision-makers to inform policy and program decisions for specific age groups.

## Additional files

Additional File 1: PRISMA checklist (DOCX 29 kb)
Additional File 2: Search strategies (DOCX 13 kb)
Additional File 3: Data collection form (PDF 91 kb)
Additional File 4: Systematic review decision-making criteria table with results (DOCX 44 kb)
Additional File 5: Systematic review report and protocol references (DOCX 63 kb)

## Abbreviations

CONSORT: Consolidated Standards of Reporting Trials
CONSORT-C: Consolidated Standards of Reporting Trials-Children
EQUATOR: Enhancing the QUality and Transparency Of health Research
PRISMA: Preferred Reporting Items in Systematic Review and Meta-Analysis
PRISMA-C: Preferred Reporting Items in Systematic Review and Meta-Analysis-Children
PRISMA-P: Preferred Reporting Items in Systematic Review and Meta-Analysis-Protocols
PRISMA-P-C: Preferred Reporting Items in Systematic Review and Meta-Analysis-Protocols for Children
SPIRIT: Standard Protocol Items: Recommendations for Interventional Trials
SPIRIT-C: Standard Protocol Items: Recommendations for Interventional Trials-Children
