# Supplementary material for: Are child-centric aspects in newborn and child health systematic review and meta-analysis protocols and reports adequately reported?—two systematic reviews
Source: Syst Rev. 2017 Mar 6;6:31. doi: 10.1186/s13643-017-0423-9 (PMC5338085; doi:10.1186/s13643-017-0423-9)
Supplement: Additional File 2: — Search strategies (DOCX 13 kb) [file 13643_2017_423_MOESM2_ESM.docx]

# Additional File 2: Search Strategies

MEDLINE/EMBASE (for systematic review protoocls)

1. exp child/

2. exp infant/

3. infant, newborn/

4. adolescent/

5. child,preschool/

6. minors/

7. puberty/

8. pediatrics/

9. exp pediatrics/

10. schools/

11. infan*.mp.

12. newborn*.mp.

13. baby*.mp.

14. babies*.mp.

15. neonat*.mp.

16. preterm*.mp.

17. postmatur*.mp.

18. child*.mp.

19. kid*.mp.

20. toddler*.mp.

21. adoles*.mp.

22. teen*.mp.

23. minor*.mp.

24. P?ediatric*.mp.

25. (young adj (adult* or person* or people*)).mp.

26. 1 or 2 or 3 or 4 or 5 or 6 or 7 or 8 or 9 or 10 or 11 or 12 or 13 or 14 or 15 or 16 or 17 or 18 or 19 or 20 or 21 or 22 or 23 or 24 or 25

27. "Protocol*".m_titl.

28. Meta-analysis.m_titl.

29. Meta-analysis.ab.

30. meta-analysis/

31. review/

32. review.mp. [mp=ti, ab, ot, nm, hw, kf, px, rx, ui, an, tn, dm, mf, dv, kw]

33. metaanalysis.mp. [mp=ti, ab, ot, nm, hw, kf, px, rx, ui, an, tn, dm, mf, dv, kw]

34. metaanalyses.mp.

35. meta-analyses.mp. [mp=ti, ab, ot, nm, hw, kf, px, rx, ui, an, tn, dm, mf, dv, kw]

36. (systematic$ adj4 (review$ or overview$)).mp. [mp=ti, ab, ot, nm, hw, kf, px, rx, ui, an, tn, dm, mf, dv, kw]

37. 28 or 29 or 30 or 31 or 32 or 33 or 34 or 35 or 36

38. 26 and 27 and 37

39. limit 38 to yr="2010 - 2014"

40. Humans/

41. Animals/

42. 40 NOT 41

43. 39 AND 42

Cochrane

1. exp child/

2. exp infant/

3. infant, newborn/

4. adolescent/

5. child, preschool/

6. minors/

7. puberty/

8. pediatrics/

9. exp pediatrics/

10. schools/

11. infan*.mp.

12. newborn* .mp.

13. baby*.mp.

14. babies*.mp.

15. neonat*.mp.

16. preterm*.mp.

17. postmatur*.mp.

18. child*.mp.

19. kid*.mp.

20. toddler*.mp.

21. adoles*.mp.

22. teen*.mp.

23. minor*.mp.

24. P?ediatric*.mp.

25. (young adj (adult* or person* or people)).mp.

26. OR\ 1-25

DARE

1. Infant*.mp.

2. Infancy.mp.

3. Newborn*.mp.

4. Baby*.mp.

5. Babies.mp.

6. Neonat*.mp.

7. Preterm*.mp.

8. Premature*.mp.

9. Postmatur*.mp.

10. Child*.mp.

11. Schoolchild*.mp.

12. School age*.mp.

13. Preschool*.mp. (978)

14. kids.mp. (7)

15. kid.mp. (0)

16. toddler*.mp. (10)

17. adoles*.mp. (2154)

18. teen*.mp. (55)

19. boy*.mp. (95)

20. girl*.mp. (94)

21. minor*.mp. (767)

22. pubert*.mp. (14)

23. pubescen*.mp. (1)

24. prepubescen*.mp. (2)

25. pediatric*.mp. (310)

26. paediatric*.mp. (550)

27. peadiatric*.mp. (0)

28. schools.mp. (262)

29. nursery school*.mp. (2)

30. kindergar*.mp. (20)

31. primary school*.mp. (31)

32. secondary school*.mp. (28)

33. elementary school*.mp. (17)

34. high school*.mp. (38)

35. OR \(1-36 ) (6146)
