# Supplementary material for: Are child-centric aspects in newborn and child health systematic review and meta-analysis protocols and reports adequately reported?—two systematic reviews
Source: Syst Rev. 2017 Mar 6;6:31. doi: 10.1186/s13643-017-0423-9 (PMC5338085; doi:10.1186/s13643-017-0423-9)
Supplement: Additional File 3: — Data collection form (PDF 91 kb) [file 13643_2017_423_MOESM3_ESM.pdf]

Systematic review of PRISMA and PRISMA-P using potential Child-centric items.

Please complete the survey below.

Thank you!

Unique ID ______________________

Accession Number ______________________

DOI ______________________

Author(s) ______________________

Title ______________________

Journal ______________________

Year ______________________

Abstract ______________________

Keywords ______________________

Reviewer

Please enter your name and date of review below:

Name of person entering data
- ◯ Mufiza
- ◯ April
- ◯ Kariym
- ◯ Chrinna

Date of review ______________________
(What is today's date)

Does this systematic review qualify for inclusion?
- ◯ Yes
- ◯ No

(Systematic reviews and meta-analyses need to be evaluating the association of an exposure/intervention with an outcome to be included in our systematic review. Diagnostic / MEthods sys review will be labelled as "No" with reason for exclusion to be discussed later.)

Reason for Exclusion ______________________
(if Unclear - Write "UNCLEAR" first, followed by reason for exclusion. If it is clearly NO, write reason for exclusion only.)

Population - What category of a pediatric population is included in this systematic review?

- ◯ Child only studies
- ◯ Mixed Child and Adult studies
- ◯ pertaining to pregnancy with outcomes assessed in newborn
- ◯ pertaining to family/community with outcomes assessed in children

(included studies with age < 18 years or average age < 21 years)

What is a publication type?

- ◯ Protocol
- ◯ Report

Is there a mention of a published protocol?

- ◯ Yes
- ◯ No

Provide a reference for the published protocol ______________________________

Is this an update/amendment of a previously published systematic review protocol/report?

- ◯ Yes
- ◯ No

Provide a reference of the previously published protocol or report. ______________________________
(if the details are not provided, write NR)

Does the full text state that the systematic review was registered with a systematic review registry?

- ◯ Yes
- ◯ No

Provide the name of the systematic review registry. ______________________________

What is the systematic review registration number? ______________________________

What is the type of systematic review?

- ◯ Cochrane
- ◯ Non-Cochrane

Name the Cochrane Collaborative Review Group. ______________________________

Is the funding source declared?

- ◯ Yes
- ◯ No

Who sponsored/funded the study? Select all that apply.

- ☐ Government
- ☐ Cochrane
- ☐ Academic or research institute
- ☐ Private (including foundations)
- ☐ Industry
- ☐ Unclear
- ☐ Other

(Select all that apply)

What is the "other" source of funding?

____

Please provide the following details of the corresponding author.

First Name ______________________________

Last Name ______________________________

Affiliated Institution ______________________________

Country of affiliation ______________________________

Email 1 ______________________________

Email 2 ______________________________

What is the design of included studies?

◯ RCTs only
◯ RCTs and other designs
◯ Non-RCTs only

What primary diagnostic category was involved in this study?

☐ Infectious disease and parasitic diseases
☐ Neoplasms (including oncology)
☐ Blood, blood forming organs, and the immune mechanism
☐ Endocrine, nutritional and metabolic disease
☐ Mental and behavioural disorders
☐ Nervous system
☐ Eye and adnexa
☐ Ear and mastoid process
☐ Circulatory system
☐ Respiratory system
☐ Digestive system
☐ Skin and subcutaneous tissue
☐ Musculoskeletal system and connective tissue
☐ Genitourinary, childbirth, and the puerperium
☐ Conditions originating in the perinatal period
☐ Congenital malformation, deformations and chromosomal abnormalities
☐ Symptoms, signs, and abnormal clinical laboratory findings not elsewhere classified
☐ Injury, poisoning, and consequences of external causes
☐ External causes of morbidity and mortality
☐ Factors influencing health status and contact with health services
☐ Oral health
☐ No Explicit Medical Condition
☐ Other

(Select all that apply. If you are unsure of the diagnostic category see http://apps.who.int/classifications/icd10/browse/2010/en)

What is the "other" diagnostic category?

____

What is the Intervention (if RCT) or Exposure (if non-RCT)?

☐ Drug / Natural Health Products
☐ Device
☐ Surgery or radiotherapy
☐ rehabilitation or psycho-social
☐ Vaccine
☐ Communication, organizational or education
☐ Prevention or screening
☐ Complex intervention (made up of more than one intervention)
☐ Other
☐ No explicit Exposure or Intervention

Please describe the complex intervention / exposure.

____

What is the "Other" intervention / exposure? or Describe if there is no explicit exposure or intervention. ________________________________

What is the comparison (classification 1)?

- ☐ Drug / Natural Health Products
- ☐ Device
- ☐ Surgery or radiotherapy
- ☐ rehabilitation or psycho-social
- ☐ Vaccine
- ☐ Communication, organizational or education
- ☐ Prevention or screening
- ☐ Complex intervention (made up of more than one intervention)
- ☐ Other
- ☐ No explicit comparison category

(Classifiction 1 for comparators)

What is the other comparison (classification 1)? or describe if there is no explicit comparison category ________________________________

What is the comparison (classification 2)?

- ☐ None
- ☐ Active
- ☐ Placebo
- ☐ Standard of care
- ☐ No treatment
- ☐ Historical control
- ☐ Other

What is the other comparison (classification 2) ________________________________

Is there a stated/defined primary outcome?

- ◯ Yes
- ◯ No

Primary outcome core area

- ☐ Death/survival
- ☐ Life impact
- ☐ Resource use/economic impact
- ☐ Pathophysiological manifestation
- ☐ Growth and development
- ☐ Other
- ☐ Unknown

What type of pathophysiological primary outcome(s) have been assessed?

- ☐ Behavioural
- ☐ Biomarkers
- ☐ Pain
- ☐ Physiological
- ☐ Psychological
- ☐ Other

(Select all that apply)

What is the "other" primary core outcome area? ________________________________

Are there stated/defined secondary outcomes?

- ◯ Yes
- ◯ No
- ◯ Unclear

Secondary outcome core area(s)

- ☐ Death/survival
- ☐ Life impact
- ☐ Resource use/economic impact
- ☐ Pathophysiological manifestation
- ☐ Growth and development
- ☐ Other

(Select all that apply)

Please select what type of pathophysiological secondary outcome(s) have been assessed

- ☐ Behavioural
- ☐ Biomarkers
- ☐ Pain
- ☐ Physiological
- ☐ Psychological
- ☐ Other

(Select all that apply)

What is the "other" secondary core outcome area? ______________________________

Comments related to outcomes

____

Does the methodological quality of included studies assessed/planned?

- ◯ Yes
- ◯ No
- ◯ Unclear

What method is used (planned) to assess the methodological quality of included studies?

- ☐ Jadad Scale
- ☐ Cochrane Risk of Bias Tool
- ☐ Newcastle_Ottawa Scale
- ☐ Other

Is the publication bias assessed/planned?

- ◯ Yes
- ◯ No
- ◯ Unclear

What method is used (planned) to assess publication bias?

- ☐ Funnel plot
- ☐ Trim and Frill Analysis
- ☐ GRADE evidence on publication bias
- ☐ Other

What other method is used to assess publication bias?

____

What method is used to assess the quality of systematic review?

- ◯ GRADE
- ◯ Other
- ◯ Not Assessed

What other method is used to assess the quality of systematic review? ______________________________

Criteria for MODIFICATION items classification as either Yes or No:
1. For an item to be recorded as a "YES", both the full red modified text AND the original text needs to be addressed. If only the black text is addressed, it is incomplete.
2. If only the red text is addressed or only partially addressed, it is incomplete.
3. If the item is not addressed at all (black OR red) then it is "NO".

ASSESSING THE SYSTEMATIC REVIEW AGAINST PRISMA-PC and PRISMA-C

TITLE

This section is applicable to PRISMA-PC and PRISMA-C.

1a/1: Identify the protocol/report as a systematic review, meta-analysis, or both for pediatric population as a focus of review, if applicabe.

(MODIFICATION for PRISMA-PC item#1a and PRISMA-C item#1)
(must be in the TITLE)

◯ Yes
◯ Incomplete
◯ No
◯ Unclear
◯ NA

If Yes/incomplete, provide justification for judgement.

____
(Yes= all red text is accounted for; Incomplete=only partial red text is accounted - if so, also comment on how you think it is incomplete)

SUPPORT/FUNDING

This section is applicable to PRISMA-PC and PRISMA-C.

Support/Funding

5a/27: Sources-Indicate sources of financial or other support for the review

5b/27: Sponsor-Provide name for the review funder and/or sponsor

5c/27: Role of sponsor/funder-Describe roles of funder(s), sponsor(s), and/or institution(s), if any, in developing the protocol

a. For each included trial in a systematic review, indicate (a plan to include) the source of financial support (such as Government, Academia or Industry), if any, in the trial(s).
(EXTENSION for PRISMA-PC item#5a-c and PRISMA-C item#27).
(Can be anywhere)

◯ Yes
◯ Incomplete
◯ No
◯ Unclear
◯ NA

If Yes/incomplete, provide justification for judgement.

____

ABSTRACT

This section is applicable to PRISMA-C only.

NA/2: Provide a structured summary including, as applicable: background; objectives; data sources; study eligibility criteria, including specifying targeted pediatric age group(s),interventions; primary and secondary outcomes; study appraisal and synthesis methods; results; limitations; conclusions and implications of key findings; systematic review registration number.
(MODIFICATION for PRISMA-C item#2)
(must be in ABSTRACT)

◯ Yes
◯ Incomplete
◯ No
◯ Unclear
◯ NA

If Yes/incomplete, provide justification for judgement.

____

a. If a systematic review includes both adults and children, describe a subgroup analysis for the targeted pediatric age group(s) in the methods and results of the abstract
(EXTENSION for PRISMA-C item#2)
(must be in ABSTRACT)

◯ Yes
◯ Incomplete
◯ No
◯ Unclear
◯ NA

If Yes/incomplete, provide justification for judgement.

____

b. THIS ITEM IS EXCLUDED - DO NOT ANSWER - Report broad search strategy related to pediatric population
(EXTENSION for PRISMA-C item#2)
(must be in ABSTRACT)

◯ Yes
◯ Incomplete
◯ No
◯ Unclear
◯ NA

If Yes/incomplete, provide justification for judgement.

____

c. Describe applicability or limits of applicability of the results of the systematic review to the targeted pediatric age group(s)
(EXTENSION for PRISMA-C item#2)
(must be in ABSTRACT)

◯ Yes
◯ Incomplete
◯ No
◯ Unclear
◯ NA

If Yes/incomplete, provide justification for judgement.

____

INTRODUCTION

This section is applicable to PRISMA-PC and PRISMA-C.

Rationale

6/3: Describe the rationale for the review in the context of what is already known.

a. In the contexts of the synthesized evidence in adults or other pediatric groups (non-targeted), explain the rationale for synthesizing evidence for the targeted pediatric age group(s). Provide hypotheses that relate to the targeted pediatric age group(s).
(EXTENSION for PRISMA-PC item#6 and PRISMA-C item#3)
(Can be anywhere)

◯ Yes
◯ Incomplete
◯ No
◯ Unclear
◯ NA

If Yes/incomplete, provide justification for judgement.

____

b. THIS ITEM IS COLLAPSED WITH NA/2-a- PLEASE DO NOT ANSWER- Describe any hypotheses that relate to the pediatric population or the pediatric subgroup(s).
(EXTENSION for PRISMA-PC item#6 and PRISMA-C item#3)
(Must be in INTRODUCTION / ABSTRACT / METHODS.)

◯ Yes
◯ Incomplete
◯ No
◯ Unclear
◯ NA

If Yes/incomplete, provide justification for judgement.

____

Objective

7/4: Provide an explicit statement of questions being addressed with reference to targeted pediatric age groups , interventions, comparisons, outcomes, and study design (PICOS).
(MODIFICATION for PRISMA-PC item#7 and PRISMA-C item#4)
(Must be in INTRODUCTION / ABSTRACT.)

◯ Yes
◯ Incomplete
◯ No
◯ Unclear
◯ NA

If Yes/incomplete, provide justification for judgement.

____

METHODS

Eligibility Criteria

This section is applicable to PRISMA-PC and PRISMA-C.

8/6: Specify study characteristics (e.g., PICOS, length of follow-up) and report characteristics (e.g., years considered, language, publication status) used as criteria for eligibility, giving rationale.

a. Justify the targeted pediatric age group(s) selected.
(EXTENSION for PRISMA-PC item#8 and PRISMA-C item#6)
(Can be anywhere.)

◯ Yes
◯ Incomplete
◯ No
◯ Unclear
◯ NA

If Yes/incomplete, provide justification for judgement.

____

b. Intervention:Justify the intervention for the targeted pediatric age group(s) addressing potential age related differences in intervention effects.
(EXTENSION for PRISMA-PC item#8 and PRISMA-C item#6)
(Can be anywhere.)

◯ Yes
◯ Incomplete
◯ No
◯ Unclear
◯ NA

If Yes/incomplete, provide justification for judgement.

____

c. Provide rationale for extrapolation or adaptation of adult intervention, if any.
(EXTENSION for PRISMA-PC item#8 and PRISMA-C item#6)
(Can be anywhere.)

◯ Yes
◯ Incomplete
◯ No
◯ Unclear
◯ NA

If Yes/incomplete, provide justification for judgement.

____

d. Comparators: Explain the choice of comparator(s) and, if applicable, evidence for the active comparator and/or standard of care for targeted pediatric age group(s).
(EXTENSION for PRISMA-PC item#8 and PRISMA-C item#6)
(Can be anywhere.)

◯ Yes
◯ Incomplete
◯ No
◯ Unclear
◯ NA

If Yes/incomplete, provide justification for judgement.

____

e. Outcomes: List and define all the primary outcomes addressed for the targeted pediatric age group(s). List and define growth and development outcomes, adverse outcomes (events), if applicable.
(EXTENSION for PRISMA-PC item#8 and PRISMA-C item#6)
(must be in METHODS.)

◯ Yes
◯ Incomplete
◯ No
◯ Unclear
◯ NA

If Yes/incomplete, provide justification for judgement.

____

f. THIS ITEM IS COLLAPSED WITH 8/6e- PLEASE DO NOT ANSWER. Outcomes: List and define short an/or long term outcomes. If there are no long term outcomes, justify.
(EXTENSION for PRISMA-PC item#8 and PRISMA-C item#6)
(must be in INTRODUCTION, METHODS or DISCUSSION)

◯ Yes
◯ Incomplete
◯ No
◯ Unclear
◯ NA

If Yes/incomplete, provide justification for judgement.

____

g. Outcomes: Explain the clinical relevance of the selected outcomes (benefits and harms) for the targeted pediatric age group(s).
(EXTENSION for PRISMA-PC item#8 and PRISMA-C item#6)
(must be in INTRODUCTION / METHODS / DISCUSSION)

◯ Yes
◯ Incomplete
◯ No
◯ Unclear
◯ NA

If Yes/incomplete, provide justification for judgement.

____

h. Outcomes: Explain the validity, feasibility and responsiveness of the outcome measures for the pre-targeted pediatric age group(s).
(EXTENSION for PRISMA-PC item#8 and PRISMA-C item#6)
(Must be in INTRO / METHODS / DISCUSSION. Need to address all three criteria (validity, feasibility and responsivenss) in order to be yes.)

◯ Yes
◯ Incomplete
◯ No
◯ Unclear
◯ NA

If Yes/incomplete, provide justification for judgement.

____

METHODS

Search

This section is applicable to PRISMA-PC and PRISMA-C.

10/8: Present full electronic search strategy for at least one database, including any limits used, such that it could be repeated.

a. Describe the search strategy and terms (including database specific MeSH terms for pediatric population) used to address the targeted pediatric age group(s).
(EXTENSION for PRISMA-PC item#10 and PRISMA-C item#8)
(Must be in METHODS / APPENDIX)

◯ Yes
◯ Incomplete
◯ No
◯ Unclear
◯ NA

If Yes/incomplete, provide justification for judgement.

____

b. THIS ITEM IS EXCLUDED. DO NOT ANSWER. Describe any tested pediatric search filters used in the systematic review, including a description of the sensitivity and precision of the search filters for retrieving child health studies.
(EXTENSION for PRISMA-PC item#10 and PRISMA-C item#8)
(must be in METHODS / DISCUSSION / APPENIDX)

◯ Yes
◯ Incomplete
◯ No
◯ Unclear
◯ NA

If Yes/incomplete, provide justification for judgement.

____

METHODS

Summary Measure

This section is applicable to PRISMA-PC and PRISMA-C.

NA/13: State the principal summary measures (e.g., risk ratio, difference in means).

a. If data were available for adult and pediatrics, provide summary measures for adult and targeted pediatric age group(s) separately.
(EXTENSION for PRISMA-C item#13)
(Must be in METHODS)

◯ Yes
◯ Incomplete
◯ No
◯ Unclear
◯ NA

If Yes/incomplete, provide justification for judgement.

____

METHODS

Synthesis of Results

This section is applicable to PRISMA-PC and PRISMA-C.

15b/14: Describe the methods of handling data and combining results of studies, if done, including measures of consistency (e.g., I2) for each meta-analysis.

a. For studies that included pediatrics and adults without a subgroup analysis of the pediatric population, describe how the data on targeted pediatric age group(s) were used in the analysis.
(EXTENSION for PRISMA-PC item#15b and PRISMA-C item#14)
(must be in METHODS.)

◯ Yes
◯ Incomplete
◯ No
◯ Unclear
◯ NA

If Yes/incomplete, provide justification for judgement.

____

METHODS

Additional Analyses

This section is applicable to PRISMA-PC and PRISMA-C.

15c/16: Describe methods of additional analyses (e.g., sensitivity or subgroup analyses for targeted pediatric age group(s) , meta-regression), if done, indicating which were pre-specified.
(MODIFICATION for PRISMA-PC item#15c and PRISMA-C item#16)
(must be in METHODS)

◯ Yes
◯ Incomplete
◯ No
◯ Unclear
◯ NA

If Yes/incomplete, provide justification for judgement.

____

RESULTS

Study Characteristics

This section is applicable to PRISMA-C only.

NA/18: For each study, present characteristics for which data were extracted, separately for targeted pediatric age group, (e.g., study size, PICOS, follow-up period) and provide the citations.

a. PLEASE EVALUATE THIS ITEM FOR NA/18 INSTEAD. Provide sample size of pediatric group and sub-groups (if applicable) for each study.
(EXTENSION for PRISMA-C item#18)
(must be in RESULTS / APPENDIX)

◯ Yes
◯ Incomplete
◯ No
◯ Unclear
◯ NA

If Yes/incomplete, provide justification for judgement.

____

RESULTS

Synthesis of Results

This section is applicable to PRISMA-C only.

NA/21: Present results of each meta-analysis done, separately for targeted pediatric age group(s), including number of events and total, confidence intervals and measures of consistency.

a. PLEASE EVALUATE THIS ITEM FOR NA/21 INSTEAD. Report the number of included studies with pediatric participants. Where applicable, report the number of events and total pediatric population on which the result synthesis is based.
(EXTENSION for PRISMA-C item#21)
(must be in RESULTS / APPENDIX)

◯ Yes
◯ Incomplete
◯ No
◯ Unclear
◯ NA

If Yes/incomplete, provide justification for judgement.

____

RESULTS

Additional Analysis

This section is applicable to PRISMA-C only.

NA/23: Give results of additional analyses, if done (e.g., sensitivity or subgroup analyses targeted pediatric age group(s), meta-regression.
(MODIFICATION for PRISMA-C item#23)
(must be in RESULTS / DISCUSSION / APPENDIX)

◯ Yes
◯ Incomplete
◯ No
◯ Unclear
◯ NA

If Yes/incomplete, provide justification for judgement.

____

DISCUSSION

Summary of Evidence

This section is applicable to PRISMA-C only.

NA/24: Summarize the main findings including the strength of evidence for each main outcome; consider their relevance to key groups (e.g., healthcare providers, users i.e., children, carer such as parents or legal guardians, and policy makers).
(MODIFICATION for PRISMA-C item#24)
(Must be in DISCUSSION / CONCLUSION. In order to be yes, must have discussed relevance to pediatric patients and/or parents)

◯ Yes
◯ Incomplete
◯ No
◯ Unclear
◯ NA

If Yes/incomplete, provide justification for judgement.

____

DISCUSSION

Limitations

This section is applicable to PRISMA-C only.

NA/24: Discuss limitations at study and outcome level (e.g., risk of bias, growth and developmental outcomes in children, and minimally important differences in children); at targeted pediatric age group(s) level (e.g. newborn, infant, under 5 years etc.);, and at review-level (e.g., incomplete retrieval of identified research, reporting bias and paucity of research in children) .
(MODIFICATION for PRISMA-C item#25)
(Must be in DISCUSSION / CONCLUSION.)

◯ Yes
◯ Incomplete
◯ No
◯ Unclear
◯ NA

If Yes/incomplete, provide justification for judgement.

____

DISCUSSION

Conclusion

This section is applicable to PRISMA-C only.

NA/26: Provide a general interpretation of the results in the context of other evidence (e.g., evidence from adult studies and pre-clinical studies). Implications for future research in practice, or policy related to the targeted pediatric age group(s).
(MODIFICATION for PRISMA-C item#26)
(Must be in DISCUSSION / CONCLUSION.)

◯ Yes
◯ Incomplete
◯ No
◯ Unclear
◯ NA

If Yes/incomplete, provide justification for judgement.

____

a. THIS ITEM IS COLLAPSED WITH ITEM NA/24. PLEASE DO NOT ANSWER. Provide the extent and limits of applicability of the synthesised evidence to the pediatric population of interest and describe the evidence and logic underlying these judgements.
(EXTENSION for PRISMA-C item#26)
(Must be in DISCUSSION / CONCLUSION.)

◯ Yes
◯ Incomplete
◯ No
◯ Unclear
◯ NA

If Yes/incomplete, provide justification for judgement.

____

b. THIS ITEM IS COLLAPSED WITH ITEM NA/26. PLEASE DO NOT ANSWER. Provide implications for research, practice, or policy related to the pediatric population where relevant (e.g., the types of research needed to address unanswered questions in the pediatric population).
(EXTENSION for PRISMA-C item#26)
(Must be in DISCUSSION / CONCLUSION.)

◯ Yes
◯ Incomplete
◯ No
◯ Unclear
◯ NA

If Yes/incomplete, provide justification for judgement.

____

Please provide any additional comments you have regarding this study/systematic review.

____
