# Supplementary material for: Are child-centric aspects in newborn and child health systematic review and meta-analysis protocols and reports adequately reported?—two systematic reviews
Source: Syst Rev. 2017 Mar 6;6:31. doi: 10.1186/s13643-017-0423-9 (PMC5338085; doi:10.1186/s13643-017-0423-9)
Supplement: Additional File 4: — Systematic review decision-making criteria table with results (DOCX 44 kb) [file 13643_2017_423_MOESM4_ESM.docx]

**Additional File 4: Systematic Review Decision-Making Criteria Table with Results**

| Section: TITLE | | **Applies to: Title only** | **PRISMA-C** | | **PRISMA-PC** | |
| --- | --- | --- | --- | --- | --- | --- |
| 1a/1: Identify the report as a systematic review, meta-analysis, or both **for pediatric population** as a focus of review, if applicable. (MODIFICATION) | | | (n) | % | (n) | % |
| Yes | - Titles needed to state that the report is a systematic review/meta-analyses, and an age group had to be present in the title. - Lax criterion here – reference to paediatric population could either be specific age range or age group (i.e. ‘child, infant, adolescent, etc.’) | | 154 | 62.1 | 55.0 | 72.4 |
| Incomplete | - If the age group *OR* the meta-analysis/systematic review designation were missing, it was therefore an ‘Incomplete’ | | 93 | 37.5 | 21 | 27.6 |
| No | - Neither systematic review/meta-analysis nor age group designation were present. | | 1 | 0.4 | 0 | 0 |
| NA | - There was no condition where NAs were allowed. | | - | - | - | - |
|  | **TOTAL (FOR APPLICABLE STUDIES)** | | 248 | | 76 | |

| Section: TITLE | | **Applies to: Title only** | **PRISMA-C** | | **PRISMA-PC** | |
| --- | --- | --- | --- | --- | --- | --- |
| 1a/1: Identify the report as a systematic review, meta-analysis, or both **for pediatric population** as a focus of review, if applicable. (MODIFICATION)  **If marked as incomplete, did it identify the report as having a focus on pediatric populations regardless of whether they identified it as a systematic review, meta-analysis, or both. (ITEM SUB-ANALYSIS).** | | | (n) | % | (n) | % |
| Yes | - An age group needed to be present in the title. - Lax criterion here – reference to paediatric population could either be specific age group or age group ‘title’ - i.e. ‘child, infant, adolescent, etc.’ | | 24 | 25.8 | 21 | 100 |
| Incomplete | - There was no condition where Incompletes were allowed. | | - | - | - | - |
| No | - If they failed to meet the ‘Yes’ condition, it was an automatic ‘No.’ | | 69 | 74.2 | 0 | 0 |
| NA | - There was no condition where NAs were allowed. | | - | - | - | - |
|  | **TOTAL (NUMBER OF INCOMPLETES)** | | 93 | | 21 | |

| Section: SUPPORT/FUNDING | | **Applies to: Any section of paper** | **PRISMA-C** | | **PRISMA-PC** | |
| --- | --- | --- | --- | --- | --- | --- |
| 27/5 Describe sources of funding for the systematic review and other support (e.g., supply of data); role of funders for the systematic review.  **a. For each included trial in a systematic review, indicate (a plan to include) the source of financial support (such as Government, Academia or Industry), if any, in the trial(s). (EXTENSION for PRISMA-PC item#5a-c and PRISMA-C item#27).** | | | (n) | % | (n) | % |
| Yes | - Had to explicitly state their intention to include info on the financial support of each trial in their systematic review, and/or include extraction form (while referencing it within the body of the text). | | 14 | 5.6 | 10 | 13.2 |
| Incomplete | - There was no condition where Incompletes were allowed. | | - | - | - | - |
| No | - If they failed to meet the ‘Yes’ condition, it was an automatic ‘No.’ | | 234 | 94.4 | 66 | 86.8 |
| NA | - There was no condition where NAs were allowed. | | - | - | - | - |
|  | **TOTAL (FOR APPLICABLE STUDIES)** | | 248 | | 76 | |

| Section: ABSTRACT | | **Applies to: Abstract only** | **PRISMA-C** | | **PRISMA-PC** |
| --- | --- | --- | --- | --- | --- |
| NA/2: Provide a structured summary including, as applicable: background; objectives; data sources; study eligibility criteria, including **specifying targeted pediatric age group(s)**,interventions; **primary and secondary outcomes**; study appraisal and synthesis methods; results; limitations; conclusions and implications of key findings; systematic review registration number. (MODIFICATION for PRISMA-C item#2) | | | (n) | % | N.A. to PRISMA-PC |
| Yes | - Needed to meet all of the criterion listed above AND at least one of the following criteria to fulfill the age group criterion: - Must either have specific ages in years, months, weeks, days, hours (i.e. ’21 years and under’) OR state very specific, commonly known age group terms, (i.e. neonates, preterm, newborn)* - ‘Child’ and ‘Children’ are not accepted unless used with another paediatric age group such as ‘adolescent’ or ‘preterm.’ (This is because it shows that the author understands that there are age-related differences) ‘Children and adults’ was not accepted because this is too broad. | | 23 | 9.3 |  |
| Incomplete | - If ≥1 criterion were missing, it was an automatic ‘Incomplete.’ | | 223 | 89.9 |  |
| No | - If they failed to meet ALL of the criterion, it was an automatic ‘No.’ | | 2 | 0.8 |  |
| NA | - There was no condition where NAs were allowed. | | - | - |  |
|  | **TOTAL (FOR APPLICABLE STUDIES)** | | 248 | |  |

*Lisa Hartling, Kristy D. M. Wittmeier, Patrina Caldwell, Hanneke van der Lee, Terry P. Klassen, Jonathan C. Craig, Martin Offringa, for the StaR Child Health Group Pediatrics Jun 2012, 129 (S3)

| Section: ABSTRACT | | **Applies to: Abstract only** | **PRISMA-C** | | **PRISMA-PC** |
| --- | --- | --- | --- | --- | --- |
| NA/2: Provide a structured summary including, as applicable: background; objectives; data sources; study eligibility criteria, including **specifying targeted pediatric age group(s)**,interventions; **primary and secondary outcomes**; study appraisal and synthesis methods; results; limitations; conclusions and implications of key findings; systematic review registration number. (MODIFICATION for PRISMA-C item#2).  **If marked as incomplete, did it provide a structured summary specifying targeted pediatric age group(s).**  **(ITEM SUB-ANALYSIS)** | | | (n) | % | N.A. to PRISMA-PC |
| Yes | - Must either have specific ages in years, months, weeks, days, hours (i.e. ’21 years old and under’) - OR state very specific, commonly known age group terms, (i.e. neonates, preterm, newborn) - ‘Child’ and ‘Children’ are not accepted unless in conjunction with another paediatric age group such as ‘adolescent’ or ‘preterm.’ (This is because it shows that the author understands that there are age-related differences) - ‘Children and adults’ was not accepted because this is too broad. | | 89 | 39.9 |  |
| Incomplete | - There was no condition where Incompletes were allowed. | | - | - |  |
| No | - If they failed to meet the ‘Yes’ condition, it was an automatic ‘No.’ | | 134 | 60.1 |  |
| NA | - There was no condition where NAs were allowed. | | - | - |  |
|  | **TOTAL (NUMBER OF INCOMPLETES)** | | 223 | |  |

| Section: ABSTRACT | | **Applies to: Abstract only** | **PRISMA-C** | | **PRISMA-PC** |
| --- | --- | --- | --- | --- | --- |
| NA/2: Provide a structured summary including, as applicable: background; objectives; data sources; study eligibility criteria, including specifying targeted pediatric age group(s),interventions; primary and secondary outcomes; study appraisal and synthesis methods; results; limitations; conclusions and implications of key findings; systematic review registration number. (MODIFICATION for PRISMA-C item#2)  **a. If a systematic review includes both adults and children, describe a subgroup analysis for the targeted pediatric age group(s) in the methods and results of the abstract (EXTENSION for PRISMA-C item#2)** | | | (n) | % | N.A. to PRISMA-PC |
| Yes | - Had to state the intention to do a subgroup analysis for children, AND present results for children separately. - This item did not apply to studies where ONLY child outcomes were observed –i.e., it applies to only mixed child/adult studies, family/community intervention studies, and maternal/child health studies where the same outcome was used for both mother and child (e.g. mother and child HIV+ status) and not presented separately (e.g. maternal morbidity vs. perinatal morbidity) | | 8 | 11.6 |  |
| Incomplete | - If the subgroup analysis was indicated/shown only in the ‘methods’ or ‘results’ section in the abstract, not both. | | 20 | 29.0 |  |
| No | - If they failed to meet the ‘Yes’ condition, it was an automatic ‘No.’ | | 41 | 59.4 |  |
| NA | - This was automatically selected for studies where ONLY child outcomes were observed. (i.e. all child-only studies and a few maternal/child health, community/child health studies) | | 179 |  |  |
|  | **TOTAL (FOR APPLICABLE STUDIES)** | | 69 | |  |

| Section: ABSTRACT | | **Applies to: Abstract only** | **PRISMA-C** | | **PRISMA-PC** |
| --- | --- | --- | --- | --- | --- |
| NA/2: Provide a structured summary including, as applicable: background; objectives; data sources; study eligibility criteria, including **specifying targeted pediatric age group(s)**,interventions; **primary and secondary outcomes**; study appraisal and synthesis methods; results; limitations; conclusions and implications of key findings; systematic review registration number. (MODIFICATION for PRISMA-C item#2)  **b. Describe applicability or limits of applicability of the results of the systematic review to the targeted pediatric age group(s) (EXTENSION for PRISMA-C item#2)** | | | (n) | % | N.A. to PRISMA-PC |
| Yes | - Had to provide explicit statement on the applicability of the results to their chosen paediatric age group, calling them by name (e.g. children, paediatric patients). Cannot generalize with terms like: ‘individuals’ or ‘people.’ - Calling for additional research in children in the face of ‘lack of evidence’ counts. | | 154 | 62.1 |  |
| Incomplete | - If they did describe the applicability/limits of applicability, but failed to explicitly link it to their age group, it was marked incomplete. | | 60 | 24.2 |  |
| No | - If they did not state applicability/limits to applicability of any kind, it was marked ‘No’ | | 34 | 13.7 |  |
| NA | - There was no condition where NAs were allowed. | | - | - |  |
|  | **TOTAL (FOR APPLICABLE STUDIES)** | | 248 | |  |

| Section: INTRODUCTION | | **Applies to: Any section of paper** | **PRISMA-C** | | **PRISMA-PC** | |
| --- | --- | --- | --- | --- | --- | --- |
| 6/3: Describe the rationale for the review in the context of what is already known.  **a. In the contexts of the synthesized evidence in adults or other pediatric groups (non-targeted), explain the rationale for synthesizing evidence for the targeted pediatric age group(s). Provide hypotheses that relate to the targeted pediatric age group(s). (EXTENSION for PRISMA-PC item#6 and PRISMA-C item#3)** | | | (n) | % | (n) | % |
| Yes | - NEED hypothesis relating to the targeted paediatric age group for a yes. - Providing an update on results identified from earlier reviews is acceptable. - Needs to discuss the relevance of evidence specifically for the targeted pediatric age group. | | 9 | 3.6 | 1 | 1.3 |
| Incomplete | - If they are missing EITHER a hypothesis or a rationale for evidence synthesis in the pediatric age group, it was marked as a ‘Incomplete’ | | 209 | 84.3 | 68 | 89.5 |
| No | - If neither rationale for evidence synthesis in the paediatric age group, nor a hypothesis was provided, it was marked ‘No’ | | 30 | 12.1 | 7 | 9.2 |
| NA | - There was no condition where NAs were allowed. | | - | - | - | - |
|  | **TOTAL (FOR APPLICABLE STUDIES)** | | 248 | | 76 | |

| Section: INTRODUCTION | | **Applies to: Any section of paper** | **PRISMA- C** | | **PRISMA-PC** | |
| --- | --- | --- | --- | --- | --- | --- |
| 6/3: Describe the rationale for the review in the context of what is already known.  a. In the contexts of the synthesized evidence in adults or other pediatric groups (non-targeted), explain the rationale for synthesizing evidence for the targeted pediatric age group(s). Provide hypotheses that relate to the targeted pediatric age group(s). (EXTENSION for PRISMA-PC item#6 and PRISMA-C item#3)  **If marked as incomplete, did it explain the rationale for evidence synthesis in the targeted pediatric age group(s)?**  **(ITEM SUB-ANALYSIS)** | | | (n) | % | (n) | % |
| Yes | - Providing an update on results identified from earlier reviews is acceptable. - Needs to discuss the relevance of evidence specifically for the targeted pediatric age group. | | 151 | 72.2 | 61 | 89.7 |
| Incomplete | - There was no condition where Incompletes were allowed. | | - | - | - | - |
| No | - If no rationale for evidence synthesis in the pediatric group were presented, it was marked ‘No’ | | 58 | 27.8 | 7 | 10.3 |
| NA | - There was no condition where NAs were allowed. | | - | - | - | - |
|  | **TOTAL** | | 209 | | 68 | |

| Section: OBJECTIVE | | | **Applies to: Introduction and/or Abstract** | **PRISMA-C** | | | | **PRISMA-PC** | | |
| --- | --- | --- | --- | --- | --- | --- | --- | --- | --- | --- |
| 7/4: Provide an explicit statement of questions being addressed with reference to **targeted pediatric age groups**, interventions, comparisons, outcomes, and study design (PICOS). (MODIFICATION for PRISMA-PC item#7 and PRISMA-C item#4) | | | | (n) | | % | | (n) | | % |
| Yes | - Needed to meet the entire list of criterion listed above. - Must either have specific ages in years, months, weeks, days, hours (i.e. ’21 years old and under’) - OR state very specific, commonly known age group terms, (i.e. neonates, preterm, newborn) - ‘Child’ and ‘Children’ are not accepted unless in conjunction with another paediatric age group such as ‘adolescent’ or ‘preterm.’ (This is because it shows that the author understands that there are age-related differences) - ‘Children and adults’ was not accepted because this is too broad. | | | 2 | | 0.8 | | 0 | | 0 |
| Incomplete | - If ≥1 criterion were missing, it was an automatic ‘Incomplete.’ | | | 234 | | 94.4 | | 74 | | 97.4 |
| No | - If they failed to meet ALL of the criterion, it was an automatic ‘No.’ | | | 12 | | 4.8 | | 2 | | 2.3 |
| NA | - There was no condition where NAs were allowed. | | | - | | - | | - | | - |
|  | **TOTAL** | | | 248 | | | | 76 | | |
| Section: OBJECTIVE | | **Applies to: Introduction and/or Abstract** | | | **PRISMA-C** | | | | **PRISMA-PC** | |
| 7/4: Provide an explicit statement of questions being addressed with reference to **targeted pediatric age groups**, interventions, comparisons, outcomes, and study design (PICOS). (MODIFICATION for PRISMA-PC item#7 and PRISMA-C item#4)  **If marked as incomplete, did it state the pediatric age group(s) within the Objective(s)? (ITEM SUB-ANALYSIS)** | | | | | (n) | | % | | (n) | % |
| Yes | - Needed to state the age in the objective. - Must either have specific ages in years, months, weeks, days, hours (i.e. ’22 years old and under’) - OR state very specific, commonly known age group terms, (i.e. neonates, preterm, newborn) - ‘Child’ and ‘Children’ are not accepted unless in conjunction with another paediatric age group such as ‘adolescent’ or ‘preterm.’ (This is because it shows that the author understands that there are age-related differences) - ‘Children and adults’ was not accepted because this is too broad. | | | | 85 | | 36.3 | | 22 | 29.7 |
| Incomplete | - There was no condition where Incompletes were allowed. | | | | - | | - | | - | - |
| No | - If they failed to state ages in the objective, it was an automatic ‘No.’ | | | | 149 | | 63.7 | | 52 | 70.3 |
| NA | - There was no condition where NAs were allowed. | | | | - | | - | | - | - |
|  | **TOTAL** | | | | 234 | | | | 74 | |

| Section: METHODS | | **Applies to: Any section of paper** | **PRISMA-C** | | **PRISMA-PC** | |
| --- | --- | --- | --- | --- | --- | --- |
| 8/6: Specify study characteristics (e.g., PICOS, length of follow-up) and report characteristics (e.g., years considered, language, publication status) used as criteria for eligibility, giving rationale.  **a. Justify the targeted pediatric age group(s) selected. (EXTENSION for PRISMA-PC item#8 and PRISMA-C item#6)** | | | (n) | % | (n) | % |
| Yes | - Had to JUSTIFY why they chose this age group – either by stating a paucity/need for evidence, or discussing the use of the exposure/intervention among children using examples from the literature. | | 184 | 74.2 | 79 | 64.5 |
| Incomplete | - If they did not adequately justify, (e.g. implied rather than explicit justification for children), it was Incomplete. | | 26 | 10.5 | 16 | 21.1 |
| No | - If they failed to meet ALL of the criterion, it was an automatic ‘No.’ | | 38 | 15.3 | 11 | 14.5 |
| NA | - There was no condition where NAs were allowed. | | - | - | - | - |
|  | **TOTAL** | | 248 | | 76 | |

| Section: METHODS | | **Applies to: Any section of paper** | **PRISMA-C** | | **PRISMA-PC** | |
| --- | --- | --- | --- | --- | --- | --- |
| 8/6: Specify study characteristics (e.g., PICOS, length of follow-up) and report characteristics (e.g., years considered, language, publication status) used as criteria for eligibility, giving rationale.  **b. Intervention: Justify the intervention for the targeted pediatric age group(s) addressing potential age related differences in intervention effects. (EXTENSION for PRISMA-PC item#8 and PRISMA-C item#6)** | | | (n) | % | (n) | % |
| Yes | - Had to JUSTIFY the why they chose this intervention – either by providing evidence for use of the intervention in children, discussing its potential use in children, or stating the paucity of evidence for its use in children. - Age-related differences was optional due to “potential” being used in the Extension item. - It is key that the authors made the justification RELEVANT to their targeted paediatric group for a yes. | | 168 | 67.7 | 42 | 55.3 |
| Incomplete | - If they did not adequately justify, (e.g. implied rather than explicit justification for children), it was Incomplete. | | 50 | 20.2 | 15 | 19.7 |
| No | - If they failed to meet ALL of the criterion, it was an automatic ‘No.’ | | 30 | 12.1 | 19 | 25.0 |
| NA | - There was no condition where NAs were allowed. | | - | - | - | - |
|  | **TOTAL** | | 248 | | 76 | |

| Section: METHODS | | **Applies to: Any section of paper** | **PRISMA-C** | | **PRISMA-PC** | |
| --- | --- | --- | --- | --- | --- | --- |
| 8/6: Specify study characteristics (e.g., PICOS, length of follow-up) and report characteristics (e.g., years considered, language, publication status) used as criteria for eligibility, giving rationale.  **c. Provide rationale for extrapolation or adaptation of adult intervention, if any. (EXTENSION for PRISMA-PC item#8 and PRISMA-C item#6)** | | | (n) | % | (n) | % |
| Yes | - Had to provide a rationale for extrapolating or adapting the intervention for children instead of adults. | | 8 | 29.6 | 3 | 16.7 |
| Incomplete | - If they did not adequately justify, (e.g. implied rather than explicit justification for children), it was Incomplete. | | 1 | 3.7 | 2 | 11.1 |
| No | - If they failed to meet ALL of the criterion, it was an automatic ‘No.’ | | 18 | 66.7 | 13 | 72.2 |
| NA | - This was an optional item, therefore reports were not penalized if they did not meet this criterion. These studies were not adaptations at all, and were therefore were marked ‘NA’ instead. | | 221 | - | 58 | - |
|  | **TOTAL** | | 248 | | 18 | |

| Section: METHODS | | **Applies to: Methods only** | **PRISMA-C** | | **PRISMA-PC** | |
| --- | --- | --- | --- | --- | --- | --- |
| 8/6: Specify study characteristics (e.g., PICOS, length of follow-up) and report characteristics (e.g., years considered, language, publication status) used as criteria for eligibility, giving rationale.  **d. Comparators: Explain the choice of comparator(s) and, if applicable, evidence for the active comparator and/or standard of care for targeted pediatric age group(s). (EXTENSION for PRISMA-PC item#8 and PRISMA-C item#6)** | | | (n) | % | (n) | % |
| Yes | - Must give a basis for choosing the comparator – giving a history of its usage as a comparator was acceptable, as was discussing the comparator as the standard of care, etc. | | 24 | 9.7 | 13 | 17.1 |
| Incomplete | - If they did not adequately justify, (e.g. merely listed the comparator), it was Incomplete. | | 3 | 1.2 | 21 | 27.6 |
| No | - If they failed to meet ALL of the criterion, it was an automatic ‘No.’ | | 221 | 89.1 | 42 | 55.6 |
| NA | - There was no condition where NAs were allowed. | | - | - | - | - |
|  | **TOTAL** | | 248 | | 76 | |

| Section: METHODS | | **Applies to: Methods only** | **PRISMA-C** | | **PRISMA-PC** | |
| --- | --- | --- | --- | --- | --- | --- |
| 8/6: Specify study characteristics (e.g., PICOS, length of follow-up) and report characteristics (e.g., years considered, language, publication status) used as criteria for eligibility, giving rationale.  **e. Outcomes: List and define all the primary outcomes addressed for the targeted pediatric age group(s). List and define growth and development outcomes, adverse outcomes (events), if applicable. (EXTENSION for PRISMA-PC item#8 and PRISMA-C item#6)** | | | (n) | % | (n) | % |
| Yes | - Had to list and define primary outcomes to be a ‘Yes’ - Growth and developmental outcomes were encouraged, but still optional due to the ‘if applicable’ statement. - In mixed studies (i.e. adult/child), there needed to be clear indication that the outcomes applied to BOTH the targeted paediatric groups and the adults. | | 102 | 41.1 | 42 | 55.3 |
| Incomplete | - If they only listed the primary outcomes, it was Incomplete. | | 74 | 29.8 | 29 | 38.2 |
| No | - If they failed to list and define the primary outcomes, it was an automatic ‘No.’ | | 72 | 29.0 | 5 | 6.6 |
| NA | - There was no condition where NAs were allowed. | | - | - | - | - |
|  | **TOTAL** | | 248 | | 76 | |

| Section: METHODS | | **Applies to: Introduction, and/or Methods, and/or Discussion** | **PRISMA-C** | | **PRISMA-PC** | |
| --- | --- | --- | --- | --- | --- | --- |
| 8/6: Specify study characteristics (e.g., PICOS, length of follow-up) and report characteristics (e.g., years considered, language, publication status) used as criteria for eligibility, giving rationale.  **f. Outcomes: Explain the clinical relevance of the selected outcomes (benefits and harms) for the targeted pediatric age group(s). (EXTENSION for PRISMA-PC item#8 and PRISMA-C item#6)** | | | (n) | % | (n) | % |
| Yes | - Had to EXPLAIN the relevance of ALL outcomes used in the systematic review/meta-analysis. - Did not have to explain the specific measurement tool to be a yes, provided they explained the relevance of the construct/biomarker that the tool is intended to measure to the targeted paediatric age group. - It was necessary that they explained the relevance to their targeted paediatric group. | | 85 | 34.3 | 22 | 28.9 |
| Incomplete | - If they did not adequately explain the relevance to their targeted paediatric group (i.e. merely listed the outcomes), or fewer than ALL of the outcomes’ clinical relevance was addressed, it was marked ‘Incomplete.’ | | 60 | 24.2 | 34 | 44.7 |
| No | - If there was no explanation of outcome relevance to the targeted paediatric age group whatsoever, it was marked ‘No.’ | | 103 | 41.5 | 20 | 26.3 |
| NA | - There was no condition where NAs were allowed. | | - | - | - | - |
|  | **TOTAL** | | 248 | | 76 | |

| Section: METHODS | | **Applies to: Introduction, and/or Methods, and/or Discussion** | **PRISMA-C** | | **PRISMA-PC** | |
| --- | --- | --- | --- | --- | --- | --- |
| 8/6: Specify study characteristics (e.g., PICOS, length of follow-up) and report characteristics (e.g., years considered, language, publication status) used as criteria for eligibility, giving rationale.  **g. Outcomes: Explain the validity, feasibility and responsiveness of the outcome measures for the pre-targeted pediatric age group(s). (EXTENSION for PRISMA-PC item#8 and PRISMA-C item#6)** | | | (n) | % | (n) | % |
| Yes | - Needed to address all three criteria (validity, feasibility, and responsiveness) to be a ‘Yes.’ | | 0 | 0 | 0 | 0 |
| Incomplete | - If 1-2 of the criterion were met, it was marked ‘Incomplete.’ | | 24 | 9.7 | 47 | 18.4 |
| No | - If none of the criteria were met, it was a no. | | 224 | 90.3 | 62 | 81.6 |
| NA | - There was no condition where NAs were allowed. | | - | - | - | - |
|  | **TOTAL** | | 248 | | 76 | |

| Section: METHODS | | **Applies to: Methods and/or Appendix** | **PRISMA-C** | | **PRISMA-PC** | |
| --- | --- | --- | --- | --- | --- | --- |
| 10/8: Present full electronic search strategy for at least one database, including any limits used, such that it could be repeated.  **a. Describe the search strategy and terms (including database specific MeSH terms for pediatric population) used to address the targeted pediatric age group(s). (EXTENSION for PRISMA-PC item#10 and PRISMA-C item#8)** | | | (n) | % | (n) | % |
| Yes | - Needed to have a search strategy with at least one search term related to the targeted paediatric age group to be a ‘Yes’ (e.g. infant, child). Mixed child/adult studies not providing these terms were marked ‘Incomplete’ since their search strategies were meant to identify literature in all ages. - Stating that they had ‘limited’ their search to ‘children’ was not acceptable on its own, since the terms used in search filters are unknown, and dependent on the database and interface used. - Pointing to an appendix was acceptable, provided that the author referenced the search strategy within the body of the Methods section. | | 109 | 44.0 | 37 | 48.7 |
| Incomplete | - If studies had no search terms but stated that they ‘limited the search strategy to children,’ or they provided a search strategy without paediatric terms, they were marked as ‘Incomplete.’ | | 115 | 46.4 | 19 | 25.0 |
| No | - If none of the criteria were met, it was a no. | | 24 | 9.7 | 20 | 26.3 |
| NA | - There was no condition where NAs were allowed. | | - | - | - | - |
|  | **TOTAL** | | 248 | | 76 | |

| Section: METHODS | | **Applies to: Methods, and/or Discussion, and/or Appendix** | | | **PRISMA-C** | | | **PRISMA-PC** | | | |
| --- | --- | --- | --- | --- | --- | --- | --- | --- | --- | --- | --- |
| NA/13: State the principal summary measures (e.g. risk ratio, difference in means).  **a. If data were available for adult and pediatrics, provide summary measures for adult and targeted pediatric age group(s) separately. (EXTENSION for PRISMA-C item#13)** | | | | | (n) | % | | (n) | | % | |
| Yes | - In systematic reviews where inclusion/exclusion criteria allowed for analysis on children and adults, they had to state their intention to present the results for adults and children separately. | | | | 2 | 2.2 | | 3 | | 12.0 | |
| Incomplete | - There was no condition where Incompletes were allowed. | | | | - | - | | - | | - | |
| No | - If they did not indicate a plan separately for adults and the targeted paediatric age group, then it was marked ‘No.’ | | | | 88 | 97.8 | | 22 | | 88.0 | |
| NA | - Child-only studies which featured no adults were marked NA by default. | | | | 158 | - | | 51 | | - | |
|  | **TOTAL** | | | | 90 | | | 25 | | | |
| Section: METHODS | | | **Applies to: Methods only** | **PRISMA-C** | | | | | **PRISMA-PC** | | |
| 15b/14: Describe the methods of handling data and combining results of studies, if done, including measures of consistency (e.g., I2) for each meta-analysis.  **a. For studies that included pediatrics and adults without a subgroup analysis of the pediatric population, describe how the data on targeted pediatric age group(s) were used in the analysis. (EXTENSION for PRISMA-PC item#15b and PRISMA-C item#14)** | | | | (n) | | | % | | (n) | | % |
| Yes | - In situations where mixed-population studies could be found, (i.e. mixed adult/child systematic reviews), they had to state a plan to deal with studies featuring data without subgroup analysis for adults and the targeted paediatric age group. | | | 0 | | | 0 | | 0 | | 0 |
| Incomplete | - There was no condition where Incompletes were allowed. | | | - | | | - | | - | | - |
| No | - If they did not indicate a plan, then it was marked ‘No.’ | | | 89 | | | 100 | | 27 | | 100 |
| NA | - Child-only studies which featured no adults were marked NA by default. | | | 159 | | | - | | 49 | | - |
|  | **TOTAL** | | | 142 | | | | | 27 | | |

| Section: METHODS | | **Applies to: Methods only** | **PRISMA-C** | | **PRISMA-PC** | |
| --- | --- | --- | --- | --- | --- | --- |
| 15c/16: Describe methods of additional analyses (e.g., sensitivity or subgroup analyses **for targeted pediatric age group(s)**, meta-regression), if done, indicating which were pre-specified. (MODIFICATION for PRISMA-PC item#15c and PRISMA-C item#16) | | | (n) | % | (n) | % |
| Yes | - Had to indicate a plan to analyze their targeted paediatric group separately from adults, or another paediatric group. This was an optional question because of the ‘if done’ statement. | | 78 | 54.9 | 36 | 61.0 |
| Incomplete | - There was no condition where Incompletes were allowed. | | - | - | - | - |
| No | - If the study performed subgroup/sensitivity analyses but did not explicitly do them with respect to their targeted age groups, it was marked as ‘Incomplete.’ | | 64 | 45.1 | 23 | 39.0 |
| NA | - Studies that did not do any sensitivity/subgroup analyses were marked as N/A due to the optional nature of the question. | | 106 | - | 17 | - |
|  | **TOTAL** | | 142 | | 59 | |

| Section: RESULTS | | **Applies to: Results and/or Appendix** | **PRISMA-C** | | **PRISMA-PC** |
| --- | --- | --- | --- | --- | --- |
| NA/18: For each study, present characteristics for which data were extracted, separately for targeted pediatric age group, (e.g, study size, PICOS, follow-up period) and provide the citations.  **a. Provide sample size of pediatric group and sub-groups (if applicable) for each study. (EXTENSION for PRISMA-C item#18)** | | | (n) | % | N.A. to PRISMA-PC |
| Yes | - Provided a sample size of the paediatric group and sub-groups for each study and mentioned the unavailability of the information. | | 170 | 72.6 |  |
| Incomplete | - Provided sample sizes, but not for every study, or not every study had this complete information. | | 26 | 11.1 |  |
| No | - If they did not provide any sample sizes whatsoever, it was marked ‘No.’ | | 38 | 16.2 |  |
| NA | - Systematic reviews that could not recover child-centric studies though children were in the inclusion criteria were marked NA due to this being a shortcoming of the literature and not the fault of the authors. | | 14 | - |  |
|  | **TOTAL** | | 234 | |  |

| Section: RESULTS | | **Applies to: Results and/or Appendix** | **PRISMA-C** | | **PRISMA-PC** |
| --- | --- | --- | --- | --- | --- |
| NA/21: Present results of each meta-analysis done, separately for targeted pediatric age group(s), including number of events and total, confidence intervals and measures of consistency.  **a. Report the numbers of included studies with pediatric participants. Where applicable, report the number of events and total pediatric population on which the result synthesis is based. (EXTENSION for PRISMA-C item#21)** | | | (n) | % | N.A. to PRISMA-PC |
| Yes | - If meta-analysis was present, they had to report the number of included studies with paediatric participants, number of events and totals, confidence intervals and measures of consistency. - Child-only studies only needed to provide the # of events and total, confidence intervals and measures of consistency for a ‘Yes’. Mixed child/adult studies needed to visually and statistically separate their targeted paediatric age group from the remaining individuals in the meta-analysis for a ‘Yes.’ | | 102 | 60.7 |  |
| Incomplete | - If meta-analysis is present, but one or more of the above criterion for ‘Yes’ were not met, it was marked ‘Incomplete.’ | | 66 | 39.3 |  |
| No | - There was no condition where ‘No’ was allowed. | | - | - |  |
| NA | - If no meta-analyses were present, it was therefore marked ‘NA.” | | 80 | - |  |
|  | **TOTAL** | | 168 | |  |
| Section: RESULTS | | **Applies to: Results, and/or Discussion, and/or Appendix** | **PRISMA-C** | | **PRISMA-PC** |
| NA/23: Give results of additional analyses, if done (e.g., sensitivity or subgroup analyses **for the targeted pediatric age group(s)**, meta-regression [see Item 16]. (MODIFICATION for PRISMA-C item#23) | | | (n) | % | N.A. to PRISMA-PC |
| Yes | - If done, the sensitivity/subgroup analysis needed to be done comparing the targeted paediatric group to the accompanying adult group or to other paediatric age groups for a ‘Yes.’ | | 47 | 54.0 |  |
| Incomplete | - There was no condition where ‘Incomplete’ was allowed. | | - | - |  |
| No | - If they performed a sensitivity/subgroup analysis but did not compare the targeted paediatric age group to other child or adult age groups, it was marked ‘Incomplete.’ | | 40 | 46.0 |  |
| NA | - If no subgroup analysis is present, or not enough information was found to do the analysis, it was marked ‘NA’ | | 161 | - |  |
|  | **TOTAL** | | 87 | |  |

| Section: DISCUSSION | | **Applies to: Discussion and/or Conclusion** | **PRISMA-C** | | **PRISMA-PC** |
| --- | --- | --- | --- | --- | --- |
| NA/24: Summarize the main findings including the strength of evidence for each main outcome; consider their relevance to key groups (e.g., healthcare providers, users **i.e., children, carer such as parents or legal guardians,** and policy makers). (MODIFICATION for PRISMA-C item#24) | | | (n) | % | N.A. to PRISMA-PC |
| Yes | - Provide a general summary of the findings for the most pertinent outcomes, and consider their relevance to the targeted pediatric group and their families. | | 178 | 74.5 |  |
| Incomplete | - If they provide a general summary of the findings, but did not consider the relevance for children and their families, it was marked ‘Incomplete.’ | | 44 | 18.4 |  |
| No | - If a discussion is offered, but they did not provide any summary of the main findings, it was marked as ‘No.’ | | 17 | 7.1 |  |
| NA | - If no studies were found and no Discussion was offered, it would be marked as ‘NA.’ If they did offer some sort of discussion, however, they were subject to the above criterion. | | 9 | - |  |
|  | **TOTAL** | | 239 | |  |

| Section: DISCUSSION | | **Applies to: Discussion and/or Conclusion** | **PRISMA-C** | | **PRISMA-PC** |
| --- | --- | --- | --- | --- | --- |
| NA/25: Discuss limitations at study and outcome level (e.g., risk of bias, **growth and developmental outcomes in children, and minimally important differences in children); at targeted pediatric age group(s) level (e.g. newborn, infant, under 5 years etc.)**, and at review-level (e.g., incomplete retrieval of identified research, reporting bias and **paucity of research in children**). (MODIFICATION for PRISMA-C item#25) | | | (n) | % | N.A. to PRISMA-PC |
| Yes | - Needed to discuss study/outcome level limitations, and review-level limitations and generalizability with respect to the targeted paediatric age groups. | | 83 | 35.8 |  |
| Incomplete | - If one of the criterion necessary for a ‘Yes’ was not met, it was therefore a ‘Incomplete.’ | | 125 | 53.9 |  |
| No | - If a discussion is offered, but limitations and generalizability was simply not discussed it was a ‘No.’ | | 24 | 10.3 |  |
| NA | - If no studies were found and no Discussion was offered, it would be marked as ‘NA.’ If they did offer some sort of discussion, however, they were subject to the above criterion. | | 16 | - |  |
|  | TOTAL | | 232 | |  |

| Section: DISCUSSION | | **Applies to: Discussion and/or Conclusion** | **PRISMA-C** | | **PRISMA-PC** |
| --- | --- | --- | --- | --- | --- |
| NA/26: Provide a general interpretation of the results in the context of other evidence (e.g., evidence from adult studies and pre-clinical studies). Implications for future research in practice, or policy **related to the targeted pediatric age group(s)**. (MODIFICATION for PRISMA-C item#26) | | | (n) | % | N.A. to PRISMA-PC |
| Yes | - Need to provide a general interpretation of the results for the targeted paediatric age group while citing previous evidence. - Must always state the implications of the research for practice and/or policy for the targeted paediatric age group. | | 107 | 43.9 |  |
| Incomplete | - If only one the two criteria is met, it was marked ‘Incomplete’ | | 120 | 49.2 |  |
| No | - If a discussion is offered, but they did not meet either of the criterion for inclusion, it was marked ‘No.’ | | 17 | 7.0 |  |
| NA | - If no studies were found and no Discussion was offered, it could be marked as ‘NA.’ If they did offer some sort of discussion, however, they were subject to the above criterion. | | 1 | - |  |
|  | **TOTAL** | | 244 | |  |
