# Supplementary material for: Are child-centric aspects in newborn and child health systematic review and meta-analysis protocols and reports adequately reported?—two systematic reviews
Source: Syst Rev. 2017 Mar 6;6:31. doi: 10.1186/s13643-017-0423-9 (PMC5338085; doi:10.1186/s13643-017-0423-9)
Supplement: Additional File 5: — Systematic review report and protocol references (DOCX 63 kb) [file 13643_2017_423_MOESM5_ESM.docx]

# Additional File 5: Systematic Review Report and Protocol References

## Included Systematic Reviews (n=248)

1. Akech S, Ledermann H, Maitland K: **Choice of fluids for resuscitation in children with severe infection and shock: systematic review**. *BMJ* 2010, **341**:c4416.

2. Alhashimi D, Fedorowicz Z, Alhashimi F, Dastgiri S: **Blood transfusions for treating acute chest syndrome in people with sickle cell disease**. *Cochrane Database Syst Rev* 2010:CD007843.

3. Beyenburg S, Stavem K, Schmidt D: **Placebo-corrected efficacy of modern antiepileptic drugs for refractory epilepsy: systematic review and meta-analysis**. *Epilepsia* 2010, **51**:7–26.

4. Browning GG, Rovers MM, Williamson I, Lous J, Burton MJ: **Grommets (ventilation tubes) for hearing loss associated with otitis media with effusion in children**. *Cochrane Database Syst Rev* 2010:CD001801.

5. Buchanan SL, Crowther CA, Levett KM, Middleton P, Morris J: **Planned early birth versus expectant management for women with preterm prelabour rupture of membranes prior to 37 weeks’ gestation for improving pregnancy outcome**. *Cochrane Database Syst Rev* 2010:CD004735.

6. Bultman E, Kuipers EJ, van der Woude CJ: **Systematic review: steroid withdrawal in anti-TNF-treated patients with inflammatory bowel disease**. *Aliment Pharmacol Ther* 2010, **32**:313–323.

7. Carlin AJ, Alfirevic Z, Gyte GM: **Interventions for treating peripartum cardiomyopathy to improve outcomes for women and babies**. *Cochrane Database Syst Rev* 2010:CD008589.

8. Carvalho DM, Salazar M, Oliveira BH de, Coutinho ESF: **Fluoride varnishes and decrease in caries incidence in preschool children: a systematic review**. *Rev Bras Epidemiol* 2010, **13**:139–149.

9. Cepeda MS, Tzortzopoulou A, Thackrey M, Hudcova J, Arora Gandhi P, Schumann R: **Adjusting the pH of lidocaine for reducing pain on injection**. *Cochrane Database Syst Rev* 2010:CD006581.

10. Ciampa PJ, Kumar D, Barkin SL, Sanders LM, Yin HS, Perrin EM, Rothman RL: **Interventions aimed at decreasing obesity in children younger than 2 years: a systematic review**. *Arch Pediatr Adolesc Med* 2010, **164**:1098–1104.

11. Courter JD, Baker WL, Nowak KS, Smogowicz LA, Desjardins LL, Coleman CI, Girotto JE: **Increased clinical failures when treating acute otitis media with macrolides: a meta-analysis**. *Ann Pharmacother* 2010, **44**:471–478.

12. Davies MW, Davis PG: **Nebulized racemic epinephrine for extubation of newborn infants**. *Cochrane Database Syst Rev* 2010:CD000506.

13. Davies MW, Woodgate PG: **Tracheal gas insufflation for the prevention of morbidity and mortality in mechanically ventilated newborn infants**. *Cochrane Database Syst Rev* 2010:CD002973.

14. Deshpande G, Rao S, Patole S, Bulsara M: **Updated Meta-analysis of Probiotics for Preventing Necrotizing Enterocolitis in Preterm Neonates**. *Pediatrics* 2010, **125**:921–930.

15. Dhulkotia JS, Ola B, Fraser R, Farrell T: **Oral hypoglycemic agents vs insulin in management of gestational diabetes: a systematic review and metaanalysis**. *Am J Obstet Gynecol* 2010, **203**:457.e1-9.

16. Görk AS, Ehrenkranz RA, Bracken MB: **Continuous infusion versus intermittent bolus doses of indomethacin for patent ductus arteriosus closure in symptomatic preterm infants**. *Cochrane Database Syst Rev* 2010:CD006071.

17. Graves PM, Deeks JJ, Demicheli V, Jefferson T: **Vaccines for preventing cholera: killed whole cell or other subunit vaccines (injected)**. *Cochrane Database Syst Rev* 2010:CD000974.

18. Henderson-Smart DJ, Davis PG: **Prophylactic methylxanthines for endotracheal extubation in preterm infants**. *Cochrane Database Syst Rev* 2010:CD000139.

19. Ho BPV, Carter M, Stephenson J: **Anger Management Using a Cognitive‐behavioural Approach for Children with Special Education Needs: A literature review and meta‐analysis**. *International Journal of Disability, Development and Education* 2010, **57**:245–265.

20. Hu J, Doucette K, Hartling L, Tjosvold L, Robinson J: **Treatment of hepatitis C in children: a systematic review**. *PLoS ONE* 2010, **5**:e11542.

21. Hunt R, Hey E: **Ethamsylate for the prevention of morbidity and mortality in preterm or very low birth weight infants**. *Cochrane Database Syst Rev* 2010:CD004343.

22. Inglis GD, Davies MW: **Prophylactic antibiotics to reduce morbidity and mortality in neonates with umbilical venous catheters**. *Cochrane Database Syst Rev* 2010, **4**:CD005251.

23. Karkhaneh M, Clark B, Ospina MB, Seida JC, Smith V, Hartling L: **Social Stories^TM^ to improve social skills in children with autism spectrum disorder A systematic review**. *Autism* 2010, **14**:641–662.

24. Lassi ZS, Haider BA, Bhutta ZA: **Zinc supplementation for the prevention of pneumonia in children aged 2 months to 59 months**. *Cochrane Database Syst Rev* 2010:CD005978.

25. Lee H, Hahn S, Lee HJ, Kim K-H: **Immunogenicity of Haemophilus influenzae Type b Conjugate Vaccines in Korean Infants: A Meta-analysis**. *J Korean Med Sci* 2010, **25**:90–96.

26. Maayan L, Vakhrusheva J, Correll CU: **Effectiveness of medications used to attenuate antipsychotic-related weight gain and metabolic abnormalities: a systematic review and meta-analysis**. *Neuropsychopharmacology* 2010, **35**:1520–1530.

27. Maclennan S, Augood C, Cash-Gibson L, Logan S, Gilbert RE: **Cisapride treatment for gastro-oesophageal reflux in children**. *Cochrane Database Syst Rev* 2010:CD002300.

28. McDonald NJ, Bara AI: **Anticholinergic therapy for chronic asthma in children over two years of age**. *Cochrane Database Syst Rev* 2010:CD003535.

29. McIntyre PG, Tosh K, McGuire W: **Caesarean section versus vaginal delivery for preventing mother to infant hepatitis C virus transmission**. *Cochrane Database Syst Rev* 2010:CD005546.

30. Osborn DA, Jeffery HE, Cole MJ: **Sedatives for opiate withdrawal in newborn infants**. *Cochrane Database Syst Rev* 2010:CD002053.

31. Patel M, Lee CK: **Polysaccharide vaccines for preventing serogroup A meningococcal meningitis**. *Cochrane Database Syst Rev* 2010:CD001093.

32. Pretell Mazzini J, Rodriguez Martin J, Andres Esteban EM: **Surgical approaches for open reduction and pinning in severely displaced supracondylar humerus fractures in children: a systematic review**. *J Child Orthop* 2010, **4**:143–152.

33. Quon BS, Fitzgerald JM, Lemière C, Shahidi N, Ducharme FM: **Increased versus stable doses of inhaled corticosteroids for exacerbations of chronic asthma in adults and children**. *Cochrane Database Syst Rev* 2010:CD007524.

34. Rodrigo G, Pollack C, Rodrigo C, Rowe BH: **Heliox for nonintubated acute asthma patients**. *Cochrane Database Syst Rev* 2010:CD002884.

35. Sharp WG, Jaquess DL, Morton JF, Herzinger CV: **Pediatric feeding disorders: a quantitative synthesis of treatment outcomes**. *Clin Child Fam Psychol Rev* 2010, **13**:348–365.

36. Shub A, Walker SP: **Planned early delivery versus expectant management for monoamniotic twins**. *Cochrane Database Syst Rev* 2010:CD008820.

37. Soltani H, Hutchon DR, Poulose TA: **Timing of prophylactic uterotonics for the third stage of labour after vaginal birth**. *Cochrane Database Syst Rev* 2010:CD006173.

38. Stolley MR, Restrepo J, Sharp LK: **Diet and physical activity in childhood cancer survivors: a review of the literature**. *Ann Behav Med* 2010, **39**:232–249.

39. Sun J, Wu X, Meng Y, Jin L: **Bupivacaine versus normal saline for relief of post-adenotonsillectomy pain in children: a meta-analysis**. *Int J Pediatr Otorhinolaryngol* 2010, **74**:369–373.

40. Theodoratou E, Al-Jilaihawi S, Woodward F, Ferguson J, Jhass A, Balliet M, Kolcic I, Sadruddin S, Duke T, Rudan I, Campbell H: **The effect of case management on childhood pneumonia mortality in developing countries**. *Int J Epidemiol* 2010, **39 Suppl 1**:i155-171.

41. van den Broek N, Dou L, Othman M, Neilson JP, Gates S, Gülmezoglu AM: **Vitamin A supplementation during pregnancy for maternal and newborn outcomes**. *Cochrane Database Syst Rev* 2010:CD008666.

42. Walsh T, Worthington HV, Glenny A-M, Appelbe P, Marinho VC, Shi X: **Fluoride toothpastes of different concentrations for preventing dental caries in children and adolescents**. *Cochrane Database Syst Rev* 2010:CD007868.

43. Wang J, Zhan P, Ouyang J, Chen B, Zhou R, Yang Y: **Unrelated donor umbilical cord blood transplantation versus unrelated donor bone marrow transplantation in adult and pediatric patients: A meta-analysis**. *Leukemia Research* 2010, **34**:1018–1022.

44. Whitworth M, Quenby S: **Prophylactic oral betamimetics for preventing preterm labour in singleton pregnancies**. *Cochrane Database Syst Rev* 2010:CD006395.

45. Abdel-Latif ME, Osborn DA: **Intratracheal Clara cell secretory protein (CCSP) administration in preterm infants with or at risk of respiratory distress syndrome**. *Cochrane Database Syst Rev* 2011:CD008308.

46. Afshari A, Brok J, Møller AM, Wetterslev J: **Inhaled nitric oxide for acute respiratory distress syndrome and acute lung injury in adults and children: a systematic review with meta-analysis and trial sequential analysis**. *Anesth Analg* 2011, **112**:1411–1421.

47. Ahmad E, Grimes DE: **The effects of self-management education for school-age children on asthma morbidity: a systematic review**. *J Sch Nurs* 2011, **27**:282–292.

48. Aikenhead A, Knai C, Lobstein T: **Effectiveness and cost-effectiveness of paediatric bariatric surgery: a systematic review**. *Clinical Obesity* 2011, **1**:12–25.

49. Ballard CK, Bricker L, Reed K, Wood L, Neilson JP: **Nutritional advice for improving outcomes in multiple pregnancies**. *Cochrane Database Syst Rev* 2011:CD008867.

50. Compalati E, Baena-Cagnani R, Penagos M, Badellino H, Braido F, Gómez RM, Canonica GW, Baena-Cagnani CE: **Systematic review on the efficacy of fexofenadine in seasonal allergic rhinitis: a meta-analysis of randomized, double-blind, placebo-controlled clinical trials**. *Int Arch Allergy Immunol* 2011, **156**:1–15.

51. De-Regil LM, Suchdev PS, Vist GE, Walleser S, Peña-Rosas JP: **Home fortification of foods with multiple micronutrient powders for health and nutrition in children under two years of age**. *Cochrane Database Syst Rev* 2011:CD008959.

52. Effa EE, Lassi ZS, Critchley JA, Garner P, Sinclair D, Olliaro PL, Bhutta ZA: **Fluoroquinolones for treating typhoid and paratyphoid fever (enteric fever)**. *Cochrane Database Syst Rev* 2011:CD004530.

53. Gao L, Mao Q, Wen D, Zhang L, Zhou X, Hui R: **The effect of beta-blocker therapy on progressive aortic dilatation in children and adolescents with Marfan’s syndrome: a meta-analysis**. *Acta Paediatr* 2011, **100**:e101-105.

54. Gillies D, Spence K: **Deep versus shallow suction of endotracheal tubes in ventilated neonates and young infants**. *Cochrane Database Syst Rev* 2011:CD003309.

55. Hanwella R, Senanayake M, de Silva V: **Comparative efficacy and acceptability of methylphenidate and atomoxetine in treatment of attention deficit hyperactivity disorder in children and adolescents: a meta-analysis**. *BMC Psychiatry* 2011, **11**:176.

56. Hellström S, Groth A, Jörgensen F, Pettersson A, Ryding M, Uhlén I, Boström KB: **Ventilation tube treatment: a systematic review of the literature**. *Otolaryngol Head Neck Surg* 2011, **145**:383–395.

57. Kelly KP, Kirschenbaum DS: **Immersion treatment of childhood and adolescent obesity: the first review of a promising intervention**. *Obes Rev* 2011, **12**:37–49.

58. Lazzerini M, Tickell D: **Antibiotics in severely malnourished children: systematic review of efficacy, safety and pharmacokinetics**. *Bulletin of the World Health Organization* 2011, **89**:594–607.

59. Lee ACC, Cousens S, Wall SN, Niermeyer S, Darmstadt GL, Carlo WA, Keenan WJ, Bhutta ZA, Gill C, Lawn JE: **Neonatal resuscitation and immediate newborn assessment and stimulation for the prevention of neonatal deaths: a systematic review, meta-analysis and Delphi estimation of mortality effect**. *BMC Public Health* 2011, **11 Suppl 3**:S12.

60. Maas T, Kaper J, Sheikh A, Knottnerus JA, Wesseling G, Dompeling E, Muris JWM, van Schayck CP: **Mono and multifaceted inhalant and/or food allergen reduction interventions for preventing asthma in children at high risk of developing asthma**. *Cochrane Database Syst Rev* 2011:CD006480.

61. McCarthy FP, Giles ML, Rowlands S, Purcell KJ, Jones CA: **Antenatal interventions for preventing the transmission of cytomegalovirus (CMV) from the mother to fetus during pregnancy and adverse outcomes in the congenitally infected infant**. *Cochrane Database Syst Rev* 2011:CD008371.

62. McQueen J, Howe TE, Allan L, Mains D, Hardy V: **Brief interventions for heavy alcohol users admitted to general hospital wards**. *Cochrane Database Syst Rev* 2011:CD005191.

63. Mei H, Pu J, Yang C, Zhang H, Zheng L, Tong Q: **Laparoscopic versus open pyeloplasty for ureteropelvic junction obstruction in children: a systematic review and meta-analysis**. *J Endourol* 2011, **25**:727–736.

64. Morag I, Ohlsson A: **Cycled light in the intensive care unit for preterm and low birth weight infants**. *Cochrane Database Syst Rev* 2011:CD006982.

65. Nagler EV, Williams G, Hodson EM, Craig JC: **Interventions for primary vesicoureteric reflux**. *Cochrane Database Syst Rev* 2011:CD001532.

66. Pillai Riddell R, Racine N, Turcotte K, Uman L, Horton R, Din Osmun L, Ahola Kohut S, Hillgrove-Stuart J, Stevens B, Lisi D: **Nonpharmacological management of procedural pain in infants and young children: an abridged Cochrane review**. *Pain Res Manag* 2011, **16**:321–330.

67. Reveiz L, Gyte GM, Cuervo LG, Casasbuenas A: **Treatments for iron-deficiency anaemia in pregnancy**. *Cochrane Database Syst Rev* 2011:CD003094.

68. Rodrigo GJ, Neffen H: **Efficacy of fluticasone furoate nasal spray vs. placebo for the treatment of ocular and nasal symptoms of allergic rhinitis: a systematic review**. *Clin Exp Allergy* 2011, **41**:160–170.

69. Rodrigo GJ, Neffen H, Castro-Rodriguez JA: **Efficacy and safety of subcutaneous omalizumab vs placebo as add-on therapy to corticosteroids for children and adults with asthma: a systematic review**. *Chest* 2011, **139**:28–35.

70. Rueda J-R, Ballesteros J, Guillen V, Tejada M-I, Solà I: **Folic acid for fragile X syndrome**. *Cochrane Database Syst Rev* 2011:CD008476.

71. Sarkhy A, Schreiber RA, Milner R, Barker CC: **Does adjuvant steroid therapy post-Kasai portoenterostomy improve the outcome of biliary atresia? A systematic review and meta-analysis**. *Can J Gastroenterol* 2011, **25**:440–444.

72. Scott P, Moss WJ, Gilani Z, Low N: **Measles vaccination in HIV-infected children: systematic review and meta-analysis of safety and immunogenicity**. *J Infect Dis* 2011, **204 Suppl 1**:S164-178.

73. Shah PS, Shah VS: **Continuous heparin infusion to prevent thrombosis and catheter occlusion in neonates with peripherally placed percutaneous central venous catheters**. *Cochrane Database Syst Rev* 2011:CD002772.

74. Shah PS, Shah VS: **Propofol for procedural sedation/anaesthesia in neonates**. *Cochrane Database Syst Rev* 2011:CD007248.

75. Slingerland M, Borghouts L: **Direct and indirect influence of physical education-based interventions on physical activity: a review**. *J Phys Act Health* 2011, **8**:866–878.

76. Song Y, Wang H, Wang B, Qi H, Rong Z, Chen H: **Atropine in ameliorating the progression of myopia in children with mild to moderate myopia: a meta-analysis of controlled clinical trials**. *J Ocul Pharmacol Ther* 2011, **27**:361–368.

77. Spinks A, Wasiak J: **Scopolamine (hyoscine) for preventing and treating motion sickness**. *Cochrane Database Syst Rev* 2011:CD002851.

78. Taylor JE, Hawley G, Flenady V, Woodgate PG: **Tracheal suctioning without disconnection in intubated ventilated neonates**. *Cochrane Database Syst Rev* 2011:CD003065.

79. Teoh L, Hurwitz M, Acworth JP, van Asperen P, Chang AB: **Treatment of obstructive sleep apnoea for chronic cough in children**. *Cochrane Database Syst Rev* 2011:CD008182.

80. Utama DP, Crowther CA: **Transplacental versus direct fetal corticosteroid treatment for accelerating fetal lung maturation where there is a risk of preterm birth**. *Cochrane Database Syst Rev* 2011:CD008981.

81. Wahi G, Parkin PC, Beyene J, Uleryk EM, Birken CS: **Effectiveness of interventions aimed at reducing screen time in children: a systematic review and meta-analysis of randomized controlled trials**. *Arch Pediatr Adolesc Med* 2011, **165**:979–986.

82. Williams G, Craig JC: **Long-term antibiotics for preventing recurrent urinary tract infection in children**. *Cochrane Database Syst Rev* 2011:CD001534.

83. Wurzel D, Marchant JM, Yerkovich ST, Upham JW, Masters IB, Chang AB: **Short courses of antibiotics for children and adults with bronchiectasis**. *Cochrane Database Syst Rev* 2011:CD008695.

84. Adeniyi FB, Young T: **Weight loss interventions for chronic asthma**. *Cochrane Database Syst Rev* 2012, **7**:CD009339.

85. Anabrees JA, Aifaleh KM: **Fluid restriction and prophylactic indomethacin in extremely low birth weight infants**. *J Clin Neonatol* 2012, **1**:1–5.

86. Baker T, Haines S, Yost J, DiClaudio S, Braun C, Holt S: **The role of family-centered therapy when used with physical or occupational therapy in children with congenital or acquired disorders**. *Physical Therapy Reviews* 2012, **17**:29–36.

87. Barnett E, Sussman S, Smith C, Rohrbach LA, Spruijt-Metz D: **Motivational Interviewing for adolescent substance use: a review of the literature**. *Addict Behav* 2012, **37**:1325–1334.

88. Bialocerkowski AE, Daly A: **Is physiotherapy effective for children with complex regional pain syndrome type 1?** *Clin J Pain* 2012, **28**:81–91.

89. Carney T, Myers B: **Effectiveness of early interventions for substance-using adolescents: findings from a systematic review and meta-analysis**. *Subst Abuse Treat Prev Policy* 2012, **7**:25.

90. Carrasco M, Volkmar FR, Bloch MH: **Pharmacologic treatment of repetitive behaviors in autism spectrum disorders: evidence of publication bias**. *Pediatrics* 2012, **129**:e1301-1310.

91. Cleland CL, Tully MA, Kee F, Cupples ME: **The effectiveness of physical activity interventions in socio-economically disadvantaged communities: a systematic review**. *Prev Med* 2012, **54**:371–380.

92. Collins J, Cheung K, Farrokhyar F, Strumas N: **Pharyngeal flap versus sphincter pharyngoplasty for the treatment of velopharyngeal insufficiency: a meta-analysis**. *J Plast Reconstr Aesthet Surg* 2012, **65**:864–868.

93. Deshpande AV, Caldwell PHY, Sureshkumar P: **Drugs for nocturnal enuresis in children (other than desmopressin and tricyclics)**. *Cochrane Database Syst Rev* 2012, **12**:CD002238.

94. Ernst E: **Craniosacral therapy: a systematic review of the clinical evidence**. *Focus on Alternative and Complementary Therapies* 2012, **17**:197–201.

95. Farah BQ, Berenguer M de F, Prado WL do, Júnior C, Gomes C, Dias RMR: **Effect of physical training on the blood pressure of adolescents with obesity**. *Revista Paulista de Pediatria* 2012, **30**:600–607.

96. Feng X, Li J, Li Y, Zhao Z, Zhao S, Wang J: **Effectiveness of TAD-anchored maxillary protraction in late mixed dentition**. *Angle Orthod* 2012, **82**:1107–1114.

97. Girard AW, Olude O: **Nutrition education and counselling provided during pregnancy: effects on maternal, neonatal and child health outcomes**. *Paediatr Perinat Epidemiol* 2012, **26 Suppl 1**:191–204.

98. Gordon M, Naidoo K, Akobeng AK, Thomas AG: **Osmotic and stimulant laxatives for the management of childhood constipation**. *Cochrane Database Syst Rev* 2012, **7**:CD009118.

99. Granados CE, Reveiz L, Uribe LG, Criollo CP: **Drugs for treating giardiasis**. *Cochrane Database Syst Rev* 2012, **12**:CD007787.

100. Gutiérrez-Castrellón P, Mayorga-Buitron JL, Bosch-Canto V, Solomon-Santibañez G, de Colsa-Ranero A: **Efficacy and safety of clarithromycin in pediatric patients with upper respiratory infections: a systematic review with meta-analysis**. *Rev Invest Clin* 2012, **64**:126–135.

101. Hetrick SE, McKenzie JE, Cox GR, Simmons MB, Merry SN: **Newer generation antidepressants for depressive disorders in children and adolescents**. *Cochrane Database Syst Rev* 2012, **11**:CD004851.

102. Huang Y-C, Li Y-C, Chen T-J: **The efficacy of intravenous immunoglobulin for the treatment of toxic epidermal necrolysis: a systematic review and meta-analysis**. *Br J Dermatol* 2012, **167**:424–432.

103. Imhoff-Kunsch B, Briggs V: **Antihelminthics in pregnancy and maternal, newborn and child health**. *Paediatr Perinat Epidemiol* 2012, **26 Suppl 1**:223–238.

104. Jefferson T, Rivetti A, Di Pietrantonj C, Demicheli V, Ferroni E: **Vaccines for preventing influenza in healthy children**. *Cochrane Database Syst Rev* 2012, **8**:CD004879.

105. Kassab M, Foster JP, Foureur M, Fowler C: **Sweet-tasting solutions for needle-related procedural pain in infants one month to one year of age**. *Cochrane Database Syst Rev* 2012, **12**:CD008411.

106. Kwok CS, Gibbs S, Bennett C, Holland R, Abbott R: **Topical treatments for cutaneous warts**. *Cochrane Database Syst Rev* 2012, **9**:CD001781.

107. Lutge EE, Wiysonge CS, Knight SE, Volmink J: **Material incentives and enablers in the management of tuberculosis**. *Cochrane Database Syst Rev* 2012, **1**:CD007952.

108. Martínez-Victoria E, Yago MD: **Omega 3 polyunsaturated fatty acids and body weight**. *Br J Nutr* 2012, **107 Suppl 2**:S107-116.

109. Meneses J, Bhandari V, Alves JG: **Nasal intermittent positive-pressure ventilation vs nasal continuous positive airway pressure for preterm infants with respiratory distress syndrome: a systematic review and meta-analysis**. *Arch Pediatr Adolesc Med* 2012, **166**:372–376.

110. Michelet D, Andreu-Gallien J, Bensalah T, Hilly J, Wood C, Nivoche Y, Mantz J, Dahmani S: **A meta-analysis of the use of nonsteroidal antiinflammatory drugs for pediatric postoperative pain**. *Anesth Analg* 2012, **114**:393–406.

111. Ng E, Taddio A, Ohlsson A: **Intravenous midazolam infusion for sedation of infants in the neonatal intensive care unit**. *Cochrane Database Syst Rev* 2012, **6**:CD002052.

112. Niccols A, Milligan K, Smith A, Sword W, Thabane L, Henderson J: **Integrated programs for mothers with substance abuse issues and their children: a systematic review of studies reporting on child outcomes**. *Child Abuse Negl* 2012, **36**:308–322.

113. Niemeier BS, Hektner JM, Enger KB: **Parent participation in weight-related health interventions for children and adolescents: a systematic review and meta-analysis**. *Prev Med* 2012, **55**:3–13.

114. Okwundu CI, Okoromah CAN, Shah PS: **Prophylactic phototherapy for preventing jaundice in preterm or low birth weight infants**. *Cochrane Database Syst Rev* 2012, **1**:CD007966.

115. Papadopoulos MA, Koumpridou EN, Vakalis ML, Papageorgiou SN: **Effectiveness of pre-surgical infant orthopedic treatment for cleft lip and palate patients: a systematic review and meta-analysis**. *Orthod Craniofac Res* 2012, **15**:207–236.

116. Parkin N, Furness S, Shah A, Thind B, Marshman Z, Glenroy G, Dyer F, Benson PE: **Extraction of primary (baby) teeth for unerupted palatally displaced permanent canine teeth in children**. *Cochrane Database Syst Rev* 2012, **12**:CD004621.

117. Qin Q, Xie Z, Shen Y, Yang S, Liu C, Huang Z, Xu J, Al J, Shen K: **Assessment of immunochemotherapy and stem cell transplantation on EBV-associated hemophagocytic lymphohistiocytosis in children: a systematic review and meta analysis**. *Eur Rev Med Pharmacol Sci* 2012, **16**:672–678.

118. Robertson-Wilson JE, Dargavel MD, Bryden PJ, Giles-Corti B: **Physical activity policies and legislation in schools: a systematic review**. *Am J Prev Med* 2012, **43**:643–649.

119. Schoonees A, Visser J, Musekiwa A, Volmink J: **Pycnogenol® (extract of French maritime pine bark) for the treatment of chronic disorders**. *Cochrane Database Syst Rev* 2012:CD008294.

120. Shah SS, Ohlsson A, Halliday HL, Shah VS: **Inhaled versus systemic corticosteroids for the treatment of chronic lung disease in ventilated very low birth weight preterm infants**. *Cochrane Database Syst Rev* 2012, **5**:CD002057.

121. Shah SS, Ohlsson A, Shah VS: **Intraventricular antibiotics for bacterial meningitis in neonates**. *Cochrane Database Syst Rev* 2012:CD004496.

122. Siegenthaler E, Munder T, Egger M: **Effect of preventive interventions in mentally ill parents on the mental health of the offspring: systematic review and meta-analysis**. *J Am Acad Child Adolesc Psychiatry* 2012, **51**:8–17.e8.

123. Strehle E-M, Gray WK, Gopisetti S, Richardson J, McGuire J, Malone S: **Can home monitoring reduce mortality in infants at increased risk of sudden infant death syndrome? A systematic review**. *Acta Paediatr* 2012, **101**:8–13.

124. Thorne-Lyman A, Fawzi WW: **Vitamin D during pregnancy and maternal, neonatal and infant health outcomes: a systematic review and meta-analysis**. *Paediatr Perinat Epidemiol* 2012, **26 Suppl 1**:75–90.

125. van Wyk GW, Hazell PL, Kohn MR, Granger RE, Walton RJ: **How oppositionality, inattention, and hyperactivity affect response to atomoxetine versus methylphenidate: a pooled meta-analysis**. *J Atten Disord* 2012, **16**:314–324.

126. van Zon A, van der Heijden GJ, van Dongen TMA, Burton MJ, Schilder AGM: **Antibiotics for otitis media with effusion in children**. *Cochrane Database Syst Rev* 2012, **9**:CD009163.

127. Venkatesh V, Khan R, Curley A, Hopewell S, Doree C, Stanworth S: **The safety and efficacy of red cell transfusions in neonates: a systematic review of randomized controlled trials**. *Br J Haematol* 2012, **158**:370–385.

128. Young L, Morgan J, McCormick FM, McGuire W: **Nutrient-enriched formula versus standard term formula for preterm infants following hospital discharge**. *Cochrane Database Syst Rev* 2012, **3**:CD004696.

129. Zhu B-H, Lv H-T, Sun L, Zhang J-M, Cao L, Jia H-L, Yan W-H, Shen Y-P: **A meta-analysis on the effect of corticosteroid therapy in Kawasaki disease**. *Eur J Pediatr* 2012, **171**:571–578.

130. Abdullah K, Kendzerska T, Shah P, Uleryk E, Parkin PC: **Efficacy of oral iron therapy in improving the developmental outcome of pre-school children with non-anaemic iron deficiency: a systematic review**. *Public Health Nutr* 2013, **16**:1497–1506.

131. Alejandria MM, Lansang MAD, Dans LF, Mantaring JB: **Intravenous immunoglobulin for treating sepsis, severe sepsis and septic shock**. *Cochrane Database Syst Rev* 2013, **9**:CD001090.

132. Almandil NB, Liu Y, Murray ML, Besag FMC, Aitchison KJ, Wong ICK: **Weight gain and other metabolic adverse effects associated with atypical antipsychotic treatment of children and adolescents: a systematic review and meta-analysis**. *Paediatr Drugs* 2013, **15**:139–150.

133. Azad MB, Coneys JG, Kozyrskyj AL, Field CJ, Ramsey CD, Becker AB, Friesen C, Abou-Setta AM, Zarychanski R: **Probiotic supplementation during pregnancy or infancy for the prevention of asthma and wheeze: systematic review and meta-analysis**. *BMJ* 2013, **347**:f6471.

134. Bond C, Woods K, Humphrey N, Symes W, Green L: **Practitioner Review: The effectiveness of solution focused brief therapy with children and families: a systematic and critical evaluation of the literature from 1990-2010**. *J Child Psychol Psychiatry* 2013, **54**:707–723.

135. Bonini M, Di Mambro C, Calderon MA, Compalati E, Schünemann H, Durham S, Canonica GW: **Beta₂-agonists for exercise-induced asthma**. *Cochrane Database Syst Rev* 2013, **10**:CD003564.

136. Boonmak S, Boonmak P, Laopaiboon M: **Deliberate hypotension with propofol under anaesthesia for functional endoscopic sinus surgery (FESS)**. *Cochrane Database Syst Rev* 2013, **6**:CD006623.

137. Case-Smith J: **Systematic review of interventions to promote social-emotional development in young children with or at risk for disability**. *Am J Occup Ther* 2013, **67**:395–404.

138. Cates CJ, Welsh EJ, Rowe BH: **Holding chambers (spacers) versus nebulisers for beta-agonist treatment of acute asthma**. *Cochrane Database Syst Rev* 2013, **9**:CD000052.

139. Cluver C, Novikova N, Hofmeyr GJ, Hall DR: **Maternal position during caesarean section for preventing maternal and neonatal complications**. *Cochrane Database Syst Rev* 2013, **3**:CD007623.

140. Danso-Appiah A, Olliaro PL, Donegan S, Sinclair D, Utzinger J: **Drugs for treating Schistosoma mansoni infection**. *Cochrane Database Syst Rev* 2013, **2**:CD000528.

141. Das JK, Salam RA, Kumar R, Bhutta ZA: **Micronutrient fortification of food and its impact on woman and child health: a systematic review**. *Syst Rev* 2013, **2**:67.

142. Dutta N, Cavanna AE: **The effectiveness of habit reversal therapy in the treatment of Tourette syndrome and other chronic tic disorders: a systematic review**. *Funct Neurol* 2013, **28**:7–12.

143. Ewing DL, Monsen JJ, Thompson EJ, Cartwright-Hatton S, Field A: **A Meta-Analysis of Transdiagnostic Cognitive Behavioural Therapy in the Treatment of Child and Young Person Anxiety Disorders**. *Behav Cogn Psychother* 2013, **43**:562–577.

144. Formosa N: **Blood glucose monitoring in children and adolescents with type 1 diabetes mellitus**. *Malta Medical Journal* 2013, **25**:31–35.

145. Furber CM, McGowan L, Bower P, Kontopantelis E, Quenby S, Lavender T: **Antenatal interventions for reducing weight in obese women for improving pregnancy outcome**. *Cochrane Database Syst Rev* 2013, **1**:CD009334.

146. Gearing RE, Schwalbe CSJ, Lee R, Hoagwood KE: **The effectiveness of booster sessions in CBT treatment for child and adolescent mood and anxiety disorders**. *Depress Anxiety* 2013, **30**:800–808.

147. Ghanizadeh A, Freeman RD, Berk M: **Efficacy and adverse effects of venlafaxine in children and adolescents with ADHD: a systematic review of non-controlled and controlled trials**. *Rev Recent Clin Trials* 2013, **8**:2–8.

148. Griffiths B, Ducharme FM: **Combined inhaled anticholinergics and short-acting beta2-agonists for initial treatment of acute asthma in children**. *Cochrane Database Syst Rev* 2013, **8**:CD000060.

149. Grynszpan O, Weiss PL (Tamar), Perez-Diaz F, Gal E: **Innovative technology-based interventions for autism spectrum disorders: A meta-analysis**. *Autism* 2013:1362361313476767.

150. Han S, Crowther CA, Moore V: **Magnesium maintenance therapy for preventing preterm birth after threatened preterm labour**. *Cochrane Database Syst Rev* 2013, **5**:CD000940.

151. Hancock EC, Cross JH: **Treatment of Lennox-Gastaut syndrome**. *Cochrane Database Syst Rev* 2013, **2**:CD003277.

152. Heinrichs J, Fritze Z, Vandermeer B, Klassen T, Curtis S: **Ultrasonographically guided peripheral intravenous cannulation of children and adults: a systematic review and meta-analysis**. *Ann Emerg Med* 2013, **61**:444–454.e1.

153. Heirs M, Suekarran S, Slack R, Light K, Gibson F, Glaser A, Hawkins M, Phillips R: **A systematic review of models of care for the follow-up of childhood cancer survivors**. *Pediatr Blood Cancer* 2013, **60**:351–356.

154. Hemilä H, Chalker E: **Vitamin C for preventing and treating the common cold**. *Cochrane Database Syst Rev* 2013, **1**:CD000980.

155. Hujoel PP: **Vitamin D and dental caries in controlled clinical trials: systematic review and meta-analysis**. *Nutr Rev* 2013, **71**:88–97.

156. Imdad A, Mullany LC, Baqui AH, El Arifeen S, Tielsch JM, Khatry SK, Shah R, Cousens S, Black RE, Bhutta ZA: **The effect of umbilical cord cleansing with chlorhexidine on omphalitis and neonatal mortality in community settings in developing countries: a meta-analysis**. *BMC Public Health* 2013, **13**:1–11.

157. Karch D, Albers L, Renner G, Lichtenauer N, von Kries R: **The efficacy of cognitive training programs in children and adolescents: a meta-analysis**. *Dtsch Arztebl Int* 2013, **110**:643–652.

158. Lands LC, Stanojevic S: **Oral non-steroidal anti-inflammatory drug therapy for lung disease in cystic fibrosis**. *Cochrane Database Syst Rev* 2013, **6**:CD001505.

159. Lee AL, Burge A, Holland AE: **Airway clearance techniques for bronchiectasis**. *Cochrane Database Syst Rev* 2013, **5**:CD008351.

160. Lin SY, Erekosima N, Kim JM, Ramanathan M, Suarez-Cuervo C, Chelladurai Y, Ward D, Segal JB: **Sublingual immunotherapy for the treatment of allergic rhinoconjunctivitis and asthma: a systematic review**. *JAMA* 2013, **309**:1278–1288.

161. Liu M-H, Sheng Y-J, Liu J-Y, Hu H-D, Zhang Q-F, Ren H: **Efficacy of telbivudine on interruption of hepatitis B virus vertical transmission: a meta-analysis**. *Ann Saudi Med* 2013, **33**:169–176.

162. Liu S, Hu P, Du X, Zhou T, Pei X: **Lactobacillus rhamnosus GG supplementation for preventing respiratory infections in children: a meta-analysis of randomized, placebo-controlled trials**. *Indian Pediatr* 2013, **50**:377–381.

163. Lobelo F, Garcia de Quevedo I, Holub CK, Nagle BJ, Arredondo EM, Barquera S, Elder JP: **School-based programs aimed at the prevention and treatment of obesity: evidence-based interventions for youth in Latin America**. *J Sch Health* 2013, **83**:668–677.

164. Lu AS, Kharrazi H, Gharghabi F, Thompson D: **A Systematic Review of Health Videogames on Childhood Obesity Prevention and Intervention**. *Games Health J* 2013, **2**:131–141.

165. Melby-Lervåg M, Hulme C: **Is working memory training effective? A meta-analytic review**. *Dev Psychol* 2013, **49**:270–291.

166. Mitchell SG, Gryczynski J, O’Grady KE, Schwartz RP: **SBIRT for adolescent drug and alcohol use: current status and future directions**. *J Subst Abuse Treat* 2013, **44**:463–472.

167. Moynihan PJ, Kelly S a. M: **Effect on Caries of Restricting Sugars Intake Systematic Review to Inform WHO Guidelines**. *J DENT RES* 2013:22034513508954.

168. Newton AS, Dong K, Mabood N, Ata N, Ali S, Gokiert R, Vandermeer B, Tjosvold L, Hartling L, Wild TC: **Brief emergency department interventions for youth who use alcohol and other drugs: a systematic review**. *Pediatr Emerg Care* 2013, **29**:673–684.

169. Okelo SO, Butz AM, Sharma R, Diette GB, Pitts SI, King TM, Linn ST, Reuben M, Chelladurai Y, Robinson KA: **Interventions to modify health care provider adherence to asthma guidelines: a systematic review**. *Pediatrics* 2013, **132**:517–534.

170. Okwundu CI, Afolabi BB: **Intramuscular versus intravenous anti-D for preventing Rhesus alloimmunization during pregnancy**. *Cochrane Database Syst Rev* 2013, **1**:CD007885.

171. Painuly N, Heun R, Painuly R, Sharan P: **Risk of cardiovascular malformations after exposure to paroxetine in pregnancy: meta-analysis**. *The Psychiatrist Online* 2013, **37**:198–203.

172. Perera R, Glasziou PP, Heneghan CJ, McLellan J, Williamson I: **Autoinflation for hearing loss associated with otitis media with effusion**. *Cochrane Database Syst Rev* 2013, **5**:CD006285.

173. Pinborg A, Wennerholm UB, Romundstad LB, Loft A, Aittomaki K, Söderström-Anttila V, Nygren KG, Hazekamp J, Bergh C: **Why do singletons conceived after assisted reproduction technology have adverse perinatal outcome? Systematic review and meta-analysis**. *Hum Reprod Update* 2013, **19**:87–104.

174. Presley JD, Chyka PA: **Intravenous lipid emulsion to reverse acute drug toxicity in pediatric patients**. *Ann Pharmacother* 2013, **47**:735–743.

175. Reiter J, Demirel N, Mendy A, Gasana J, Vieira ER, Colin AA, Quizon A, Forno E: **Macrolides for the long-term management of asthma--a meta-analysis of randomized clinical trials**. *Allergy* 2013, **68**:1040–1049.

176. Riggin L, Ramakrishna J, Sommer DD, Koren G: **A 2013 updated systematic review & meta-analysis of 36 randomized controlled trials; no apparent effects of non steroidal anti-inflammatory agents on the risk of bleeding after tonsillectomy**. *Clin Otolaryngol* 2013, **38**:115–129.

177. Romano M, Minozzi S, Zaina F, Saltikov JB, Chockalingam N, Kotwicki T, Hennes AM, Negrini S: **Exercises for adolescent idiopathic scoliosis: a Cochrane systematic review**. *Spine* 2013, **38**:E883-893.

178. Selph SS, Bougatsos C, Blazina I, Nelson HD: **Behavioral interventions and counseling to prevent child abuse and neglect: a systematic review to update the US Preventive services task force recommendation**. *Ann Intern Med* 2013, **158**:179–190.

179. Smith J, Thyoka M: **What role does laparoscopy play in the diagnosis and immediate treatment of infants with necrotizing enterocolitis?** *J Laparoendosc Adv Surg Tech A* 2013, **23**:397–401.

180. Temple AR, Temple BR, Kuffner EK: **Dosing and Antipyretic Efficacy of Oral Acetaminophen in Children**. *Clinical Therapeutics* 2013, **35**:1361–1375.e45.

181. Thiruvenkatachari B, Harrison JE, Worthington HV, O’Brien KD: **Orthodontic treatment for prominent upper front teeth (Class II malocclusion) in children**. *Cochrane Database Syst Rev* 2013, **11**:CD003452.

182. Thompson M, Dana T, Bougatsos C, Blazina I, Norris SL: **Screening for hypertension in children and adolescents to prevent cardiovascular disease**. *Pediatrics* 2013, **131**:490–525.

183. Vereenooghe L, Langdon PE: **Psychological therapies for people with intellectual disabilities: a systematic review and meta-analysis**. *Res Dev Disabil* 2013, **34**:4085–4102.

184. Watson J, McGuire W: **Transpyloric versus gastric tube feeding for preterm infants**. *Cochrane Database Syst Rev* 2013, **2**:CD003487.

185. Wight D, Fullerton D: **A review of interventions with parents to promote the sexual health of their children**. *J Adolesc Health* 2013, **52**:4–27.

186. Wood TJ, Quinn KM, Farrokhyar F, Deheshi B, Corbett T, Ghert MA: **Local control of extra-abdominal desmoid tumors: systematic review and meta-analysis**. *Rare Tumors* 2013, **5**.

187. Zou C, Su G, Wu Y, Lu F, Mao W, Liu X: **Astragalus in the prevention of upper respiratory tract infection in children with nephrotic syndrome: evidence-based clinical practice**. *Evid Based Complement Alternat Med* 2013, **2013**:352130.

188. Alves Galvão MG, Rocha Crispino Santos MA, Alves da Cunha AJL: **Amantadine and rimantadine for influenza A in children and the elderly**. *Cochrane Database Syst Rev* 2014, **11**:CD002745.

189. Alves Galvão MG, Rocha Crispino Santos MA, Alves da Cunha AJL: **Antibiotics for preventing suppurative complications from undifferentiated acute respiratory infections in children under five years of age**. *Cochrane Database Syst Rev* 2014, **2**:CD007880.

190. Bee P, Bower P, Byford S, Churchill R, Calam R, Stallard P, Pryjmachuk S, Berzins K, Cary M, Wan M, Abel K: **The clinical effectiveness, cost-effectiveness and acceptability of community-based interventions aimed at improving or maintaining quality of life in children of parents with serious mental illness: a systematic review**. *Health Technol Assess* 2014, **18**:1–250.

191. Beggs S, Wong ZH, Kaul S, Ogden KJ, Walters JAE: **High-flow nasal cannula therapy for infants with bronchiolitis**. *Cochrane Database Syst Rev* 2014, **1**:CD009609.

192. Bell EF, Acarregui MJ: **Restricted versus liberal water intake for preventing morbidity and mortality in preterm infants**. *Cochrane Database Syst Rev* 2014:CD000503.

193. Budden A, Chen LJY, Henry A: **High-dose versus low-dose oxytocin infusion regimens for induction of labour at term**. *Cochrane Database Syst Rev* 2014, **10**:CD009701.

194. Chalk CH, Benstead TJ, Keezer M: **Medical treatment for botulism**. *Cochrane Database Syst Rev* 2014, **2**:CD008123.

195. Chan DK, Parikh SR: **Perioperative ketorolac increases post-tonsillectomy hemorrhage in adults but not children**. *Laryngoscope* 2014, **124**:1789–1793.

196. Chen Y-P, Howard AM: **Effects of robotic therapy on upper-extremity function in children with cerebral palsy: A systematic review**. *Dev Neurorehabil* 2014, **19**:64–71.

197. Chen Y-P, Pope S, Tyler D, Warren GL: **Effectiveness of constraint-induced movement therapy on upper-extremity function in children with cerebral palsy: a systematic review and meta-analysis of randomized controlled trials**. *Clin Rehabil* 2014, **28**:939–953.

198. Cheney G, Schlösser A, Nash P, Glover L: **Targeted group-based interventions in schools to promote emotional well-being: a systematic review**. *Clin Child Psychol Psychiatry* 2014, **19**:412–438.

199. Chong LY, Clarkson JE, Dobbyn-Ross L, Bhakta S: **Slow-release fluoride devices for the control of dental decay**. *Cochrane Database Syst Rev* 2014, **11**:CD005101.

200. Conde-Agudelo A, Díaz-Rossello JL: **Kangaroo mother care to reduce morbidity and mortality in low birthweight infants**. *Cochrane Database Syst Rev* 2014, **4**:CD002771.

201. Cundy TP, Harling L, Marcus HJ, Athanasiou T, Darzi AW: **Meta analysis of robot-assisted versus conventional laparoscopic fundoplication in children**. *Journal of Pediatric Surgery* 2014, **49**:646–652.

202. Dewar R, Love S, Johnston LM: **Exercise interventions improve postural control in children with cerebral palsy: a systematic review**. *Dev Med Child Neurol* 2014, **57**:504–520.

203. El Marroun H, White T, Verhulst FC, Tiemeier H: **Maternal use of antidepressant or anxiolytic medication during pregnancy and childhood neurodevelopmental outcomes: a systematic review**. *Eur Child Adolesc Psychiatry* 2014, **23**:973–992.

204. Farley RC, Jardine LA, Hough JL: **Strategies for the withdrawal of humidified high flow nasal cannulae (HHFNC) in preterm infants**. *Cochrane Database Syst Rev* 2014:CD011079.

205. Foster BA, Tom D, Hill V: **Hypotonic versus isotonic fluids in hospitalized children: a systematic review and meta-analysis**. *J Pediatr* 2014, **165**:163–169.e2.

206. Fryda CM, Hulme PA: **School-based childhood sexual abuse prevention programs: an integrative review**. *J Sch Nurs* 2014, **31**:167–182.

207. Gadomski AM, Scribani MB: **Bronchodilators for bronchiolitis**. *Cochrane Database Syst Rev* 2014, **6**:CD001266.

208. Gawade PL, Hudson MM, Kaste SC, Neglia JP, Constine LS, Robison LL, Ness KK: **A systematic review of dental late effects in survivors of childhood cancer**. *Pediatr Blood Cancer* 2014, **61**:407–416.

209. Habbu SG, Krishnappa P: **Effectiveness of oral health education in children – a systematic review of current evidence (2005–2011)**. *Int Dent J* 2014, **65**:57–64.

210. Harvey A, Baker L, Williams K: **Non-surgical prevention and management of scoliosis for children with Duchenne muscular dystrophy: what is the evidence?** *J Paediatr Child Health* 2014, **50**:E3-9.

211. Jiao J, Li Q, Chu J, Zeng W, Yang M, Zhu S: **Effect of n−3 PUFA supplementation on cognitive function throughout the life span from infancy to old age: a systematic review and meta-analysis of randomized controlled trials**. *Am J Clin Nutr* 2014:ajcn.095315.

212. Kelly MJ, Trikalinos TA, Dahabreh IJ, Gianferante M, Parsons SK: **Cranial radiation for pediatric T-lineage acute lymphoblastic leukemia: a systematic review and meta-analysis**. *Am J Hematol* 2014, **89**:992–997.

213. Louis D, More K, Oberoi S, Shah PS: **Intravenous immunoglobulin in isoimmune haemolytic disease of newborn: an updated systematic review and meta-analysis**. *Arch Dis Child Fetal Neonatal Ed* 2014, **99**:F325-331.

214. MacLaren Chorney J, Twycross A, Mifflin K, Archibald K: **Can we improve parents’ management of their children’s postoperative pain at home?** *Pain Res Manag* 2014, **19**:e115-123.

215. Maitra S, Baidya DK, Bhattacharjee S, Khanna P: **Evaluation of i-gel^TM^ airway in children: a meta-analysis**. *Paediatr Anaesth* 2014, **24**:1072–1079.

216. Manias E, Kinney S, Cranswick N, Williams A, Borrott N: **Interventions to reduce medication errors in pediatric intensive care**. *Ann Pharmacother* 2014, **48**:1313–1331.

217. Mbeye NM, ter Kuile FO, Davies M-A, Phiri KS, Egger M, Wandeler G, IeDEA-Southern Africa: **Cotrimoxazole prophylactic treatment prevents malaria in children in sub-Saharan Africa: systematic review and meta-analysis**. *Trop Med Int Health* 2014, **19**:1057–1067.

218. Minozzi S, Amato L, Bellisario C, Davoli M: **Detoxification treatments for opiate dependent adolescents**. *Cochrane Database Syst Rev* 2014:CD006749.

219. Nauta J, van Mechelen W, Otten RHJ, Verhagen EALM: **A systematic review on the effectiveness of school and community-based injury prevention programmes on risk behaviour and injury risk in 8-12 year old children**. *J Sci Med Sport* 2014, **17**:165–172.

220. Ndikom CM, Fawole B, Ilesanmi RE: **Extra fluids for breastfeeding mothers for increasing milk production**. *Cochrane Database Syst Rev* 2014, **6**:CD008758.

221. Normansell R, Walker S, Milan SJ, Walters EH, Nair P: **Omalizumab for asthma in adults and children**. *Cochrane Database Syst Rev* 2014, **1**:CD003559.

222. Okuzawa N, Kline E, Fuertes J, Negi S, Reeves G, Himelhoch S, Schiffman J: **Psychotherapy for adolescents and young adults at high risk for psychosis: a systematic review**. *Early Intervention in Psychiatry* 2014, **8**:307–322.

223. Opiyo N, Molyneux E, Sinclair D, Garner P, English M: **Immediate fluid management of children with severe febrile illness and signs of impaired circulation in low-income settings: a contextualised systematic review**. *BMJ Open* 2014, **4**:e004934.

224. Pakhale S, Mulpuru S, Verheij TJM, Kochen MM, Rohde GGU, Bjerre LM: **Antibiotics for community-acquired pneumonia in adult outpatients**. *Cochrane Database Syst Rev* 2014, **10**:CD002109.

225. Park HY, Maitra K, Achon J, Loyola E, Rincón M: **Effects of early intervention on mental or neuromusculoskeletal and movement-related functions in children born low birthweight or preterm: a meta-analysis**. *Am J Occup Ther* 2014, **68**:268–276.

226. Patel A, Li L, Anand A: **Systematic review: functional outcomes and complications of intramedullary nailing versus plate fixation for both-bone diaphyseal forearm fractures in children**. *Injury* 2014, **45**:1135–1143.

227. Power RF, Murphy JF: **Tongue-tie and frenotomy in infants with breastfeeding difficulties: achieving a balance**. *Archives of Disease in Childhood* 2014, **100**:489–494.

228. Psaila K, Foster JP, Richards R, Jeffery HE: **Non-nutritive sucking for gastro-oesophageal reflux disease in preterm and low birth weight infants**. *Cochrane Database Syst Rev* 2014, **10**:CD009817.

229. Quirk H, Blake H, Tennyson R, Randell TL, Glazebrook C: **Physical activity interventions in children and young people with Type 1 diabetes mellitus: a systematic review with meta-analysis**. *Diabet Med* 2014, **31**:1163–1173.

230. Reyes-Portillo JA, Mufson L, Greenhill LL, Gould MS, Fisher PW, Tarlow N, Rynn MA: **Web-Based Interventions for Youth Internalizing Problems: A Systematic Review**. *Journal of the American Academy of Child & Adolescent Psychiatry* 2014, **53**:1254–1270.e5.

231. Rosen LJ, Myers V, Hovell M, Zucker D, Noach MB: **Meta-analysis of Parental Protection of Children From Tobacco Smoke Exposure**. *Pediatrics* 2014:peds.2013-0958.

232. Saeterdal I, Lewin S, Austvoll-Dahlgren A, Glenton C, Munabi-Babigumira S: **Interventions aimed at communities to inform and/or educate about early childhood vaccination**. *Cochrane Database Syst Rev* 2014, **11**:CD010232.

233. Schulzke SM, Kaempfen S, Patole SK: **Pentoxifylline for the prevention of bronchopulmonary dysplasia in preterm infants**. *Cochrane Database Syst Rev* 2014:CD010018.

234. Skelton JA, Irby MB, Geiger AM: **A systematic review of satisfaction and pediatric obesity treatment: new avenues for addressing attrition**. *J Healthc Qual* 2014, **36**:5–22.

235. Slopen N, McLaughlin KA, Shonkoff JP: **Interventions to Improve Cortisol Regulation in Children: A Systematic Review**. *Pediatrics* 2014:peds.2013-1632.

236. Soares NMM, Leão AS, Santos JR, Monteiro GR, dos Santos JR, Thomazzi SM, Silva RJ dos S: **Systematic review shows only few reliable studies of physical activity intervention in adolescents**. *ScientificWorldJournal* 2014, **2014**:206478.

237. Stratakis N, Gielen M, Chatzi L, Zeegers MP: **Effect of maternal n-3 long-chain polyunsaturated fatty acid supplementation during pregnancy and/or lactation on adiposity in childhood: a systematic review and meta-analysis of randomized controlled trials**. *Eur J Clin Nutr* 2014, **68**:1277–1287.

238. Tong Y, Ren H, Ding X, Jin S, Chen Z, Li Q: **Analgesic effect and adverse events of dexmedetomidine as additive for pediatric caudal anesthesia: a meta-analysis**. *Paediatr Anaesth* 2014, **24**:1224–1230.

239. Ung D, Selles R, Small BJ, Storch EA: **A Systematic Review and Meta-Analysis of Cognitive-Behavioral Therapy for Anxiety in Youth with High-Functioning Autism Spectrum Disorders**. *Child Psychiatry Hum Dev* 2014, **46**:533–547.

240. Valentino PL, Church PC, Shah PS, Beyene J, Griffiths AM, Feldman BM, Kamath BM: **Hepatotoxicity caused by methotrexate therapy in children with inflammatory bowel disease: a systematic review and meta-analysis**. *Inflamm Bowel Dis* 2014, **20**:47–59.

241. Vasques C, Magalhães P, Cortinhas A, Mota P, Leitão J, Lopes VP: **Effects of intervention programs on child and adolescent BMI: A meta-analysis study**. *J Phys Act Health* 2014, **11**:426–444.

242. Vloemans AFPM, Hermans MHE, van der Wal MBA, Liebregts J, Middelkoop E: **Optimal treatment of partial thickness burns in children: A systematic review**. *Burns* 2014, **40**:177–190.

243. Vuorio A, Kuoppala J, Kovanen PT, Humphries SE, Tonstad S, Wiegman A, Drogari E: **Statins for children with familial hypercholesterolemia**. *Cochrane Database Syst Rev* 2014, **7**:CD006401.

244. Weitz M, Strahm B, Meerpohl JJ, Bassler D: **Extracorporeal photopheresis versus standard treatment for acute graft-versus-host disease after haematopoietic stem cell transplantation in paediatric patients**. *Cochrane Database Syst Rev* 2014, **2**:CD009759.

245. Yap P, Utzinger J, Hattendorf J, Steinmann P: **Influence of nutrition on infection and re-infection with soil-transmitted helminths: a systematic review**. *Parasites & Vectors* 2014, **7**:229.

246. Zhang X, Kurtz M, Lee S-Y, Liu H: **Early Intervention for Preterm Infants and Their Mothers: A Systematic Review**. *J Perinat Neonatal Nurs* 2014.

247. Zhao J-G, Ding N, Huang W-J, Wang J, Shang J, Zhang P: **Interventions for treating simple bone cysts in the long bones of children**. *Cochrane Database Syst Rev* 2014, **9**:CD010847.

248. Zhou Y–., Li H–., Zhu L–., Liu J–.: **Impact of cesarean section on placental transfusion and iron-related hematological indices in term neonates: a systematic review and meta-analysis**. *Placenta* 2014, **35**:1–8.

## Included Systematic Review Protocols (n=76)

1. Fedorowicz Z, Pedrazzi V, Oliveira-Neto JM, Shinkai R, Radhi A: **Chlorhexidine treatment for the prevention of dental caries in children and adolescents (Protocol)**. *Cochrane Database Syst Rev* 2010:CD008457.

2. Parsons C, Davenport M: **Glucocorticosteroids for infants with biliary atresia following Kasai portoenterostomy (Protocol)**. *Cochrane Database Syst Rev* 2010:CD008735.

3. Dempsey E, Barrington KJ, Pammi M, Ryan A: **Formal resuscitation training courses for reducing mortality and morbidity in newborn infants (Protocol)**. *Cochrane Database Syst Rev* 2011:CD009106.

4. Irving GJ, Holden J, Pope D: **Postexposure vaccines for hepatitis A (Protocol)**. *Cochrane Database Syst Rev* 2011:CD009050.

5. Kantharajah M, Zaman FD, Samra D, Gunturu MN, Del Mar CB, van Driel ML: **Immunoglobulin treatment for respiratory syncytial virus infection (Protocol)**. *Cochrane Database Syst Rev* 2011:CD009417.

6. Lloyd C, Logan S, McHugh C, Humphreys G, Parker S, Beswick D, Beswick M, Rogers M, Thompson-Coon J, Morris C, Wyatt K: **Sleep positioning for children with cerebral palsy (Protocol)**. *Cochrane Database Syst Rev* 2011:CD009257.

7. Adekanmbi V, Uthman OA, Egbewale BE: **Routine viral load monitoring versus standard care for reducing morbidity and mortality in adolescent and adult patients living with HIV on antiretroviral therapy (ART) (Protocol)**. *Cochrane Database Syst Rev* 2012:CD009588.

8. Bambra CL, Hillier FC, Moore HJ, Summerbell CD: **Tackling inequalities in obesity: a protocol for a systematic review of the effectiveness of public health interventions at reducing socioeconomic inequalities in obesity amongst children**. *Syst Rev* 2012, **1**:16.

9. Bélard S, Kurth F, Ramharter M: **Paediatric Formulations of Artemisinin-Combination Therapies for Treating Uncomplicated Malaria in Children (Protocol)**. *Cochrane Database Syst Rev* 2012:CD009568.

10. Crowley AE, Grivell RM, Dodd JM: **Sealing procedures for preterm prelabour rupture of membranes (Protocol)**. *Cochrane Database Syst Rev* 2012:CD010218.

11. Crowther CA, Aghajafari F, Askie LM, Asztalos EV, Brocklehurst P, Bubner TK, Doyle LW, Dutta S, Garite TJ, Guinn DA, Hallman M, Hannah ME, Hardy P, Maurel K, Mazumder P, McEvoy C, Middleton PF, Murphy KE, Peltoniemi OM, Peters D, Sullivan L, Thom EA, Voysey M, Wapner RJ, Yelland L, Zhang S: **Repeat prenatal corticosteroid prior to preterm birth: a systematic review and individual participant data meta-analysis for the PRECISE study group (prenatal repeat corticosteroid international IPD study group: assessing the effects using the best level of evidence) - study protocol**. *Syst Rev* 2012, **1**:12.

12. Gertler P, Tate RL, Cameron ID: **Non-pharmacological interventions for depression in adults and children with traumatic brain injury (Protocol)**. *Cochrane Database Syst Rev* 2012:CD009871.

13. Gunaratne AW, Makrides M, Collins CT: **Maternal prenatal and/or postnatal n-3 fish oil supplementation for preventing allergies in early childhood (Protocol)**. *Cochrane Database Syst Rev* 2012:CD010085.

14. Hogan M-E, Shah VS, Smith RW, Yiu A, Taddio A: **Glucose for the management of procedural pain in neonates (Protocol)**. *Cochrane Database Syst Rev* 2012:CD009721.

15. Kallapiran GK, Mammen P, Tharyan P, Russell P: **Atomoxetine for Attention Deficit Hyperactivity Disorder (ADHD) in children and adolescents (Protocol)**. *Cochrane Database Syst Rev* 2012:CD009804.

16. Liu Q, Long Q, Garner P: **Growth monitoring and promotion (GMP) for children in low and middle income countries (Protocol)**. *Cochrane Database Syst Rev* 2012:CD010102.

17. Pearson M, Chilton R, Woods HB, Wyatt K, Ford T, Abraham C, Anderson R: **Implementing health promotion in schools: protocol for a realist systematic review of research and experience in the United Kingdom (UK)**. *Syst Rev* 2012, **1**:48.

18. Piotrowska PJ, Stride CB, Rowe R: **Social gradients in child and adolescent antisocial behavior: a systematic review protocol**. *Syst Rev* 2012, **1**:38.

19. Prutsky GJ, Domecq JP, Elraiyah T, Wang Z, Grohskopf LA, Prokop LJ, Montori VM, Murad MH: **Influenza vaccines licensed in the United States in healthy children: a systematic review and network meta-analysis (Protocol)**. *Syst Rev* 2012, **1**:65.

20. Pulman J, Bromley R, Adab N, Greenhalgh J, McKay AJ, Tudur Smith C, Dickson RC, Marson AG: **Treatment for epilepsy in pregnancy: congenital malformation outcomes in the child (Protocol)**. *Cochrane Database Syst Rev* 2012:CD010224.

21. Tricco AC, Chit A, Hallett D, Soobiah C, Meier G, Chen M, Tashkandi M, Bauch C, Loeb M: **Effect of influenza vaccines against mismatched strains: a systematic review protocol**. *Syst Rev* 2012, **1**:35.

22. van As JW, van den Berg H, van Dalen EC: **Platinum-induced hearing loss after treatment for childhood cancer (Protocol)**. *Cochrane Database Syst Rev* 2012:CD010181.

23. Anabrees J, Shah VS, AlOsaimi A, AlFaleh K: **Glycerin laxatives for prevention or treatment of feeding intolerance in very low birth weight infants (Protocol)**. *Cochrane Database Syst Rev* 2013:CD010464.

24. Ashfaq K, Yusuf BJ, Jilani AZ, Owais SS, Yahaya I, Chen Y-F, Kinsey SE: **Stem cell transplantation for high risk acute lymphoblastic leukaemia in paediatric patients in first remission (Protocol)**. *Cochrane Database Syst Rev* 2013:CD010348.

25. Badeaux J, Hawley D: **Effectiveness of intravenous Tranexamic Acid (TXA) administration in managing perioperative blood loss in patients undergoing spine surgery: a systematic review protocol**. *The JBI Database of Systematic Reviews and Implementation Reports* 2013, **11**:123–131.

26. Breik O, Tivey D, Umapathysivam K, Anderson P: **Mandibular distraction osteogenesis in the management of airway obstruction in children: A Systematic Review Protocol**. *The JBI Database of Systematic Reviews and Implementation Reports* 2013, **11**:16.

27. Degraeuwe PL, Jaspers GJ, Robertson NJ, Kessels AG: **Magnetic resonance spectroscopy as a prognostic marker in neonatal hypoxic-ischemic encephalopathy: a study protocol for an individual patient data meta-analysis**. *Syst Rev* 2013, **2**:96.

28. Eames AJ, Grivell RM, Deussen AR, Hague W, Dodd JM: **Metformin for women who are obese during pregnancy for improving maternal and infant outcomes (Protocol)**. *Cochrane Database Syst Rev* 2013:CD010564.

29. Fang Y, Jv Y, Zhang H, Liu W, Hui X: **Radiation therapy for diffuse brainstem glioma in children and young adults (Protocol)**. *Cochrane Database Syst Rev* 2013:CD010439.

30. Fitzpatrick EM, Stevens A, Garritty C, Moher D: **The effects of sign language on spoken language acquisition in children with hearing loss: a systematic review protocol**. *Syst Rev* 2013, **2**:108.

31. Grimbly C, Odenbach J, Vandermeer B, Forgie S, Curtis S: **Parenteral and oral antibiotic duration for treatment of pediatric osteomyelitis: a systematic review protocol**. *Syst Rev* 2013, **2**:92.

32. Hines S, Munday J, Kynoch K: **Effectiveness of nurse-led preoperative assessment services for elective surgery: a systematic review update protocol**. *The JBI Database of Systematic Reviews and Implementation Reports* 2013, **11**:73.

33. Kurmis R, Aromataris E, Greenwood J: **The effectiveness of trace element supplementation following severe burn injury: A systematic review protocol**. *The JBI Database of Systematic Reviews and Implementation Reports* 2013, **11**:44.

34. Lakshman R, Mazarello Paes V, Hesketh K, O’Malley C, Moore H, Ong K, Griffin S, van Sluijs E, Summerbell C: **Protocol for systematic reviews of determinants/correlates of obesity-related dietary and physical activity behaviors in young children (preschool 0 to 6 years): evidence mapping and syntheses**. *Syst Rev* 2013, **2**:28.

35. Li Q, Zhou D, Li Q, Wu B, Zhou H: **Levetiracetam monotherapy for epilepsy (Protocol)**. *Cochrane Database Syst Rev* 2013:CD010634.

36. Machingaidze S, Rehfuess E, von Kries R, Hussey GD, Wiysonge CS: **Understanding interventions for improving routine immunization coverage in children in low- and middle-income countries: a systematic review protocol**. *Syst Rev* 2013, **2**:106.

37. Mohamed S, Adi Y, AlFaleh K: **Oxandrolone for growth-hormone treated children and adolescents with Turner syndrome (Protocol)**. *Cochrane Database Syst Rev* 2013:CD010736.

38. Nisar MI, Jehan F, Shafiq Y, Lassi ZS, Zaidi AK: **Conjugate vaccines for preventing Haemophilus influenzae type b infections in children under 5 years of age (Protocol)**. *Cochrane Database Syst Rev* 2013:CD010377.

39. Paradies Y, Priest N, Ben J, Truong M, Gupta A, Pieterse A, Kelaher M, Gee G: **Racism as a determinant of health: a protocol for conducting a systematic review and meta-analysis**. *Syst Rev* 2013, **2**:85.

40. Qureshi R, Jadotte Y: **The association between prenatal exposure to environmental tobacco smoke and childhood obesity: a systematic review protocol**. *The JBI Database of Systematic Reviews and Implementation Reports* 2013, **11**:225.

41. Ramsey LS, Watkins L, Engel ME: **Health education interventions to raise awareness of rheumatic fever: a systematic review protocol**. *Syst Rev* 2013, **2**:58.

42. Sandy-Hodgetts K, Watts R: **Effectiveness of topical negative pressure/closed incision management in the prevention of post-surgical wound complications: a systematic review protocol**. *The JBI Database of Systematic Reviews and Implementation Reports* 2013, **11**:12.

43. Thummathai K, Lasuka D, Sethabouppha H, Chanprasit C: **Internet-Based Depression Prevention in Adolescents: A Systematic Review Protocol**. *The JBI Database of Systematic Reviews and Implementation Reports* 2013, **11**:197.

44. Tricco AC, Soobiah C, Antony J, Hemmelgarn B, Moher D, Hutton B, Straus SE: **Safety of serotonin (5-HT3) receptor antagonists in patients undergoing surgery and chemotherapy: protocol for a systematic review and network meta-analysis**. *Syst Rev* 2013, **2**:46.

45. Wyatt KD, Prutsky Lopez G, Domecq Garces JP, Erwin P, Brinkman WB, Montori VM, LeBlanc A: **Study protocol: a systematic review of pediatric shared decision making**. *Syst Rev* 2013, **2**:48.

46. Abdelgadir IS, Phillips RS, Moncreiff MP, Lumsden JL: **Video-laryngoscopy versus direct laryngoscopy for tracheal intubation in children (excluding neonates) (Protocol)**. *Cochrane Database Syst Rev* 2014:CD011413.

47. Abdullahi LH, Kagina BM, Cassidy T, Adebayo EF, Wiysonge CS, Hussey GD: **Knowledge, attitudes and practices on adolescent vaccination among parents, teachers and adolescents in Africa: a systematic review protocol**. *Syst Rev* 2014, **3**:100.

48. Adetokunboh OO, Schoonees A, Wiysonge CS: **Antiviral efficacy and safety of abacavir-containing combination antiretroviral therapy as first-line treatment of HIV-infected children and adolescents: a systematic review protocol**. *Syst Rev* 2014, **3**:87.

49. Brown HE, Atkin AJ, Panter J, Corder K, Wong G, Chinapaw MJM, Sluijs E van: **Family-based interventions to increase physical activity in children: a meta-analysis and realist synthesis protocol**. *BMJ Open* 2014, **4**:e005439.

50. Chong J, Haran C, Asher I: **Intermittent inhaled corticosteroid therapy versus placebo for persistent asthma in children and adults (Protocol)**. *Cochrane Database Syst Rev* 2014:CD011032.

51. Cundy WJ, Umapathysivam K, Lockwood C, Freeman B: **Effectiveness and cost effectiveness of anterior versus posterior approaches for thoracolumbar fusion in adolescent idiopathic scoliosis: a systematic review protocol**. *The JBI Database of Systematic Reviews and Implementation Reports* 2014, **12**:47.

52. Davies R, Davis D, Pearce M, Wong N: **The effect of waterbirth on neonatal mortality and morbidity: a systematic review protocol**. *The JBI Database of Systematic Reviews and Implementation Reports* 2014, **12**:89.

53. Druyts E, Eapen S, Wu P, Thorlund K: **The risk of elevated prolactin levels in pediatric patients exposed to antipsychotics for the treatment of schizophrenia and schizophrenia spectrum disorders: protocol for a systematic review and meta-analysis**. *Syst Rev* 2014, **3**:116.

54. Feyissa GT, Lockwood C, Munn Z: **The effectiveness of home-based HIV counselling and testing on reducing stigma and risky sexual behavior among adults and adolescents: a systematic review protocol**. *The JBI Database of Systematic Reviews and Implementation Reports* 2014, **12**:157.

55. Freeman JA, Marsden J, Rapson R, Kent B: **The clinical effectiveness and personal experience of supported standing for children with cerebral palsy: a comprehensive systematic review protocol**. *The JBI Database of Systematic Reviews and Implementation Reports* 2014, **12**:101.

56. Gebremedhin AT, Gesesew HA, Demissie TD, Kerie MW, Sudhakar M: **Khat Chewing and Risky Sexual Behavior in Sub Saharan Africa: A systematic Review Protocol**. *The JBI Database of Systematic Reviews and Implementation Reports* 2014, **11**:59.

57. Han H, Corbin R, Godfrey CM, Leung L, Chen J, Lee MS, Zhang T, Feng L: **The safety and effectiveness of bitter melon (momordica charantia) as an alternative to traditional hypoglycemic agents for the control of fasting blood sugar in patients with type 2 diabetes mellitus: a systematic review protocol**. *The JBI Database of Systematic Reviews and Implementation Reports* 2014, **11**:17.

58. Hayes N, Umapathysivam K, Foster B: **Interventions for treating growth plate fractures of the distal femur: a systematic review protocol**. *The JBI Database of Systematic Reviews and Implementation Reports* 2014, **12**:92.

59. Hocking JA, Pearson A, McNeil J: **Physiotherapy to improve gross motor skills in people with intellectual disability: a systematic review protocol**. *The JBI Database of Systematic Reviews and Implementation Reports* 2014, **11**:94.

60. Kalan R, Wiysonge CS, Ramafuthole T, Allie K, Ebrahim F, Engel ME: **Mobile phone text messaging for improving the uptake of vaccinations: a systematic review protocol**. *BMJ Open* 2014, **4**:e005130.

61. Kurz ES, Davis D: **Routine culture based screening versus risk based management for the prevention of early onset group B streptococcus disease in the neonate: a systematic review protocol**. *The JBI Database of Systematic Reviews and Implementation Reports* 2014, **12**:62.

62. Manspeaker S, Kelley H, Riddle D: **Treatment of exertional rhabdomyolysis among athletes: a systematic review protocol**. *The JBI Database of Systematic Reviews and Implementation Reports* 2014, **12**:113.

63. Morgan JE, Stewart L, Phillips RS: **Protocol for a systematic review of reductions in therapy for children with low-risk febrile neutropenia**. *Syst Rev* 2014, **3**:119.

64. Ogunlesi TA, Lesi FE: **Prophylactic intravenous calcium therapy for exchange blood transfusion in the newborn (Protocol)**. *Cochrane Database Syst Rev* 2014:CD011048.

65. Paavilainen E, Flinck A: **The effectiveness of methods designed to identify child maltreatment in social and health care: a systematic review protocol**. *The JBI Database of Systematic Reviews and Implementation Reports* 2014, **12**:90.

66. Pagsberg AK, Tarp S, Glintborg D, Stenstrøm AD, Fink-Jensen A, Correll CU, Christensen R: **Antipsychotic treatment for children and adolescents with schizophrenia spectrum disorders: protocol for a network meta-analysis of randomised trials**. *BMJ Open* 2014, **4**:e005708.

67. Pfadenhauer LM, Burns J, Rohwer A, Rehfuess EA: **A protocol for a systematic review of the effectiveness of interventions to reduce exposure to lead through consumer products and drinking water**. *Syst Rev* 2014, **3**:36.

68. Purgato M, Gross AL, Jordans MJ, Jong JT de, Barbui C, Tol W: **Psychosocial interventions for children exposed to traumatic events in low- and middle-income countries: study protocol of an individual patient data meta-analysis**. *Syst Rev* 2014, **3**:34.

69. Quinlan J, Tu MT, Langlois EV, Kapoor M, Ziegler D, Fahmi H, Zunzunegui MV: **Protocol for a systematic review of the association between chronic stress during the life course and telomere length**. *Syst Rev* 2014, **3**:40.

70. Riitano D, Pearson A: **The effectiveness of interventions designed to improve academic outcomes in children and adolescents in out-of-home care: a systematic review protocol**. *The JBI Database of Systematic Reviews and Implementation Reports* 2014, **12**:13.

71. Stevens A, Hamel C, Singh K, Ansari MT, Myers E, Ziegler P, Hutton B, Sharma A, Bjerre LM, Fenton S, Gow R, Hadjiyannakis S, O’Hara K, Pound C, Salewski E, Shrier I, Willows N, Moher D, Tremblay M: **Do sugar-sweetened beverages cause adverse health outcomes in children? A systematic review protocol**. *Syst Rev* 2014, **3**:96.

72. Sung V, Cabana MD, D’Amico F, Deshpande G, Dupont C, Indrio F, Mentula S, Partty A, Savino F, Szajewska H, Tancredi D: **Lactobacillus reuteri DSM 17938 for managing infant colic: protocol for an individual participant data meta-analysis**. *BMJ Open* 2014, **4**:e006475.

73. Tesfay K, Abera M, Demissie TD, Kerie MW, Morankar S: **Efficacy of narrative exposure therapy in the treatment of children and adolescent patients with posttraumatic stress disorder: a systematic review protocol**. *The JBI Database of Systematic Reviews and Implementation Reports* 2014, **12**:64.

74. Townshend K, Jordan Z, Peters MD, Tsey K: **The effectiveness of Mindful Parenting programs in promoting parents’ and children’s wellbeing: a systematic review protocol**. *The JBI Database of Systematic Reviews and Implementation Reports* 2014, **12**:184.

75. van der Heijden LB, Feskens EJ, Janse AJ: **Maintenance interventions for overweight or obese children and adolescents who participated in a treatment program: study protocol for a systematic review**. *Syst Rev* 2014, **3**:111.

76. Young MM, Stevens A, Galipeau J, Pirie T, Garritty C, Singh K, Yazdi F, Golfam M, Pratt M, Turner L, Porath-Waller A, Arratoon C, Haley N, Leslie K, Reardon R, Sproule B, Grimshaw J, Moher D: **Effectiveness of brief interventions as part of the Screening, Brief Intervention and Referral to Treatment (SBIRT) model for reducing the nonmedical use of psychoactive substances: a systematic review**. *Syst Rev* 2014, **3**:50.
